# Intestinal microbiota profiles associated with low and high residual feed intake in chickens across two geographical locations

**DOI:** 10.1371/journal.pone.0187766

**Published:** 2017-11-15

**Authors:** Sina-Catherine Siegerstetter, Stephan Schmitz-Esser, Elizabeth Magowan, Stefanie Urimare Wetzels, Qendrim Zebeli, Peadar G. Lawlor, Niamh E. O'Connell, Barbara U. Metzler-Zebeli

**Affiliations:** 1 Institute of Animal Nutrition and Functional Plant Compounds, Department for Farm Animals and Veterinary Public Health, University of Veterinary Medicine, Vienna, Austria; 2 Research Cluster “Animal Gut Health”, Department for Farm Animals and Veterinary Public Health, University of Veterinary Medicine, Vienna, Austria; 3 Institute of Milk Hygiene, Milk Technology and Food Science, Department for Farm Animals and Veterinary Public Health, University of Veterinary Medicine, Vienna, Austria; 4 Agri-Food and Biosciences Institute, Agriculture Branch, Large Park, Co. Down, Hillsborough, Northern Ireland, United Kingdom; 5 Teagasc Pig Development Department, Animal & Grassland Research & Innovation Centre, Moorepark, Fermoy, Co. Cork, Ireland; 6 Institute for Global Food Security, Queen's University Belfast, University Road Belfast, Belfast, Northern Ireland, United Kingdom; Wageningen University, NETHERLANDS

## Abstract

Intestinal microbe-host interactions can affect the feed efficiency (FE) of chickens. As inconsistent findings for FE-associated bacterial taxa were reported across studies, the present objective was to identify whether bacterial profiles and predicted metabolic functions that were associated with residual feed intake (RFI) and performance traits in female and male chickens were consistent across two different geographical locations. At six weeks of life, the microbiota in ileal, cecal and fecal samples of low (n = 34) and high (n = 35) RFI chickens were investigated by sequencing the V3-5 region of the 16S rRNA gene. Location-associated differences in α-diversity and relative abundances of several phyla and genera were detected. RFI-associated bacterial abundances were found at the phylum and genus level, but differed among the three intestinal sites and between males and females. Correlation analysis confirmed that, of the taxonomically classifiable bacteria, *Lactobacillus* (5% relative abundance) and two *Lactobacillus crispatus*-OTUs in feces were indicative for high RFI in females (*P* < 0.05). In males, *Ruminococcus* in cecal digesta (3.1% relative abundance) and *Dorea* in feces (<0.1% relative abundance) were best indicative for low RFI, whereas *Acinetobacter* in feces (<1.5% relative abundance) related to high RFI (*P* < 0.05). Predicted metabolic functions in feces of males confirmed compositional relationships as functions related to amino acid, fatty acid and vitamin metabolism correlated with low RFI, whereas an increasing abundance of bacterial signaling and interaction (i.e. cellular antigens) genes correlated with high RFI (*P* < 0.05). In conclusion, RFI-associated bacterial profiles could be identified across different geographical locations. Results indicated that consortia of low-abundance taxa in the ileum, ceca and feces may play a role for FE in chickens, whereby only bacterial FE-associations found in ileal and cecal digesta may serve as useful targets for dietary strategies.

## Introduction

Chicken’s intestinal microbiota are an important “metabolic organ” which plays a vital role in feed digestibility, nutrient absorption and immune competence [1]. Differences in microbial energy-harvesting from feed can influence energy retention, weight gain and hence chicken’s feed efficiency (FE) [1,2]. An improvement in FE reduces the feed costs and concurrently the environmental impact of broiler production [3]. Within one chicken population from the same breed, a considerable variation in FE can be found [4]. Therefore, elucidating the FE-associated intestinal microbiota composition may allow for the characterization of an optimal microbial profile for good FE. Correspondingly, bacteria belonging to *Bacteroides*, *Enterobacteriaceae*, *Clostridium*, *Ruminococcus*, *Faecalibacterium* and *Lactobacillus* have been previously positively or negatively associated with FE in chickens [5–8]. However, the high hygiene levels in modern commercial hatcheries have an unwanted side effect of causing highly variable bacterial colonization of chicken’s intestine [9]. This may be one reason for the inconsistent findings for cecal and fecal microbial profiles associated with good FE among studies [2,5,7,8] and the batch-to-batch variability within one study [7]. Other sources of variation are the dietary composition, the chicken line used and the encountered environmental microbes [2,5,7,10]. Bacteria associated with good FE, however, should be detectable across different chicken batches within the same rearing environment, but also across multiple production settings, irrespective of the origin of the chickens and dietary effects. If unique bacterial FE-associations can be characterized across batches and geographical environments, those can serve as targets for dietary approaches in the future to improve chicken’s FE. Also, previous research mainly focused on male chickens [2,5,7] and adult hens [8],whereas FE-related bacterial profiles were hardly investigated simultaneously in broiler chickens of both sexes.

So far, most studies on the FE-associated intestinal microbiota in chickens have been conducted using feed conversion ratio (FCR) or apparent metabolizable energy (AME) as metrics for FE [2,5,7]. In being independent of production traits (e.g., body weight gain), the residual feed intake (RFI) is another metric of choice to investigate the biological mechanisms contributing to varying FE in livestock animals including poultry, pigs and cattle [11,12]. The objective of this study was therefore to identify whether bacterial profiles and predicted metabolic functions that were associated with RFI or performance traits in female and male chickens were consistent across two geographical locations. This was based on the hypothesis that the ileal, cecal and fecal microbiota in chickens of good FE raised in different environments would be characterized by mutual FE-associated bacteria and microbial functions. Because batch-to-batch variation in FE-associated bacterial profiles were reported before [2,5,7,8], we designed the chicken experiments to be carried out in three batches at each geographical location in order to reduce the impact of batch-associated differences when identifying bacterial profiles across the two locations. Other sources of variation in the FE-related microbial profiles among studies may be associated with the methods of DNA extraction, sequencing and bioinformatic analyses of the samples [13]. In order to reduce the analytical bias, ileal and cecal digesta and fecal samples underwent the same analytical procedures and were processed together at one geographical location in the present study.

## Materials and methods

### Animals, housing and experimental design

To reduce the impact of experimental procedures on the study outcome, two chicken experiments using common protocols comprising the experimental design, diet formulations, data and sample collection were conducted at two different geographical locations [L1: Institute of Animal Nutrition and Functional Plant Compounds, University of Veterinary Medicine Vienna, Austria; and L2: Agri-Food and Biosciences Institute (AFBI), Hillsborough, Northern Ireland]. In order to account for the previously reported variation in the intestinal microbiota composition among chicken batches [2,5,7,8], each of the two experiments was planned to consist of three consecutive replicate batches. The two experiments were run in parallel at the two geographical locations within a six month period. In Austria, the animal procedures including animal handling and treatment were approved by the institutional ethics committee of the University of Veterinary Medicine Vienna and the Austrian national authority according to paragraph 26 of Law for Animal Experiments, Tierversuchsgesetz 2012 –TVG 2012 (GZ 68.205/0131-II/3b/2013). In Northern Ireland, animal procedures were conducted in line with the requirements of the ‘Animals (Scientific Procedures) Act 1986’ (PPL 2781, Department of Health, Social Services and Public Safety, Northern Ireland, UK) and were approved by AFBI’s internal ethics committee.

In order to mimic commercial production conditions, different local hatcheries delivered the chickens to the two geographical locations and the feed was prepared at local feed mills according to the same dietary specifications (S1 Table). A total of 157 (n = 78 females, n = 79 males) and 192 (n = 96 females, n = 96 males) day-old Cobb 500FF broiler chickens were used at L1 and L2, respectively. Within each replicate batch, equal numbers of females and males, except for one batch with one more male at L1, were used. Day-old chickens were group-housed for six days and then randomly allocated to individual cages on day 7 of life until day 42 of life. Chickens had free access to starter (day 1–10), grower (day 11–21) and finisher (day 22–42) corn-soybean meal based diets (S1 Table) and demineralized water. Diets were free of anti-microbial growth promoters and coccidiostats. At each location, starter, grower and finisher diets for the three replicate batches came from the same batch of commercially prepared crumbles (starter diet) and pellets (grower and finisher diets), which were stored in cool (<15°C) and dry conditions for a duration of no longer than six months.

### Determination of FE and selection of chickens

Feed leftovers and spills were collected before recording feed intake on days 14, 21, 28, 35, 36 and 38 of life. Chickens were weighed accordingly upon arrival, weekly and on the last experimental day. In the present study, we used the RFI to rank the chickens according to their FE [14]. The final RFI at the two geographical locations were determined two days apart due to the slower growth of the chickens at L2 than those at L1. For this, total FI (TFI), total BW gain (TBWG) and metabolic mid weight were calculated between days 7 and 36 of life and days 7 and 38 of life at L1 and L2, respectively. Regression analysis was performed for each batch and geographical location individually. A nonlinear mixed model (SAS Stat Inc., version 9.2; Cary, NC, USA) was used to estimate chicken’s RFI value as the residual according to the following equations [14]. The mid metabolic weight (MMW) was calculated as MMW = {[BW at d 7 of life (g) + BW at d 36 or 38 of life, respectively (g)]/2}^0.75^. The RFI was calculated as RFI (g) = TFI − (a_1_ + b_1_ × MMW + b_2_ × TBWG), in which a_1_ is the intercept and b_1_ and b_2_ are partial regression coefficients of MMW and TBWG on TFI, respectively. In each replicate, it was aimed to select the three chickens with the lowest RFI (low RFI chickens) and the three chickens with the highest RFI (high RFI chickens), separately for female and male chickens. A total of 34 low RFI and 35 high RFI chickens were finally selected at both geographical locations (L1, n = 9/sex and RFI; L2, n = 6 low RFI females, n = 8 high RFI females, and n = 10 low RFI males, n = 9 high RFI males). For those chickens, the RFI as well as TFI and TBWG between days 7 and 36 of life will be presented. In addition, the individual FCR value of the low and high RFI chickens was calculated.

### Sample collection

Fresh fecal samples for microbiota analysis were collected on day 36 of life at both geographical locations. To facilitate the collection of feces, parchment paper was laid out on the tray under each cage. The gastrointestinal origin of the chicken feces determines the fecal bacterial composition [15]; therefore, feces of a paste-like texture without the uric acid-containing white part were predominantly collected. Within 5 to 10 min after defecation, feces were aseptically collected, placed into sterile 2-ml cryotubes (Sarstedt, Nümbrecht, Germany), snap frozen in liquid N_2_ and stored at -80°C until DNA extraction. Selected chickens were humanely killed on days 37 to 42 of life with an overdose of sodium pentobarbital (450 mg/kg, Release, WTD-Wirtschaftsgenossenschaft Deutscher Tierärzte, Garbsen, Germany) by i.v. injection into the caudal tibial vein from day 37 of life with three to six chickens per day, whereas at L2 selected chickens were sacrificed on days 41 and 42 of life. After exsanguination, the abdominal cavity was opened and the whole gastrointestinal tract was removed. Digesta from the ileum (end of mesentery arteries to ileo-cecal junction) and both ceca were aseptically collected, homogenized, placed into sterile 2-ml cryotubes (Sarstedt), snap-frozen in liquid N_2_ and stored at -80°C until further analysis. Before homogenization, the content of both ceca was pooled together.

### DNA extraction

The DNA was extracted from all intestinal and fecal samples of both geographical locations together at L1. Total DNA was extracted from 250 mg of fecal, ileal and cecal samples using the PowerSoil DNA extraction kit (MoBio Laboratories Inc., Carlsbad, CA, USA) with some modifications [16, 17]. After addition of buffer C1, a heating step at 70°C for 10 min was included. The DNA concentration was measured using the Qubit 2.0 Fluorometer (Life Technologies, Carlsbad, CA, USA) with the Qubit dsDNA HS Assay Kit (Life Technologies). The identical DNA sample was used for 16S rRNA gene amplicon sequencing and qPCR analysis of total bacteria. For the qPCR, DNA sample volumes were adjusted to achieve similar DNA concentrations across samples to avoid an impact of the DNA concentration on the amplification results.

### 16S rRNA gene amplicon sequencing

The 16S rRNA gene was amplified using the primer set 341F (CCTACGGGRSGCAGCAG) and 909R (TTTCAGYCTTGCGRCCGTAC) targeting the V3-5 hypervariable regions of the 16S rRNA gene to generate an approximate amplicon size of 568 bp. The 16S rRNA gene PCR, library preparation as well as DNA sequencing of samples was performed by a commercial provider (Microsynth AG, Balgach, Switzerland). The 16S rRNA gene was amplified using the KAPA HiFi HotStart PCR Kit (Roche, Baden, Switzerland) including a high-fidelity DNA polymerase. Libraries were constructed by ligating sequencing adapters and indices onto purified PCR products using the Nextera XT sample preparation kit (Illumina Inc., San Diego, CA, USA) according to the manufacturer’s protocol. For each of the libraries, equimolar amounts were pooled and sequenced on an Illumina MiSeq Personal Sequencer using a 300 bp read length paired-end protocol. All sample libraries were sequenced in the same sequencing run. The resultant overlapping paired-end reads were demultiplexed, trimmed using cutadapt (http://code.google.com/p/cutadapt/) and stitched using Fast Length Adjustment of SHort reads (FLASH; http://www.cbcb.umd.edu/software/flash) [18] by Microsynth.

Further sequence processing was performed using the Quantitative Insights Into Microbial Ecology (QIIME) package, version 1.9.1 [19]. Samples from all three intestinal sites and both geographical locations were analyzed together at L1. Fastq files were quality trimmed using the “split_libraries_fastq”script for non-multiplexed Illumina fastq data with the phred offset 33. The UCHIME method using the 64-bit version of USEARCH and GOLD database (drive5.com) was used to detect and remove chimeric sequences [20,21]. Open-reference operational taxonomic unit (OTU) picking was done at 97% similarity level using UCLUST [20]. OTU taxonomy was assigned against the Greengenes database and QIIME 1.9.1 defaults (http://qiime.org/home_static/dataFiles.html) [22]. Rare OTUs containing less than 10 sequences were removed. Community metrics (coverage, α- and β-diversity) analysis was done in QIIME. For α- and β-diversity analyses a rarefaction depth of 10,000 sequences per sample was used, excluding samples with fewer reads. Beta-diversity was determined using unweighted and weighted UniFrac distance [23,24]. Unweighted UniFrac is a qualitative measurement, taking into account the presence/absence of taxa and measures the distance between two communities by calculating the fraction of the branch length in a phylogenetic tree that is unique to any of the two communities [23]. Weighted UniFrac is a quantitative measurement accounting for differences in the relative abundance of OTUs between different communities by weighting the branches in the phylogenetic tree based on the relative abundance of sequences in the communities [23,25]. The resulting distance matrices were visualized using principal coordinates analysis (PCoA). To compare the overall intestinal microbial community structures of the individual chickens between the two geographical locations and intestinal sites, Venn diagrams, including all OTUs generated by the OTU picking step, were calculated using the software mothur (version 1.34.0) [26]. Individual high abundance OTUs that correlated with RFI were additionally classified by using the Greengenes 16S rRNA gene database (http://greengenes.lbl.gov/). Raw sequencing data are available in NCBI’s BioProject SRA database under accession no. PRJNA375981.

### Quantitative PCR

Quantification of 16S rRNA gene copies of total bacteria in all DNA samples was performed on a Stratagene Mx3000P QPCR System (Agilent Technologies, Santa Clara, CA) using the Fast-Plus EvaGreen Master Mix with Low ROX (Biotium, Hayward, CA, USA Technologies) and the primer set 341-357F and 518-534R in 20 μl reaction mixtures at L1 [16,17]. Each standard and sample reaction contained 10 μl of master mix, forward and reverse primers (62.5 pmol) and 1 ng of DNA template. The amplification program included an initial denaturation step at 95°C for 5 min, followed by 40 cycles of 95°C for 15 s, primer annealing at 60°C for 30 s and elongation at 72°C for 30 s. Fluorescence was measured at the last step of each cycle. The dissociation of PCR products were monitored by slow heating with an increment of 0.5°C/s from 55 to 95°C to determine the specificity of the amplification. Correct PCR product length was additionally verified by horizontal gel electrophoresis. Amplification efficiency was calculated according to: E = -1 + 10^−1/slope^. The standard was created from the purified and quantified PCR products generated by standard PCR using DNA from chicken intestinal digesta and feces of the present experiment and the total bacterial primer set [16,17]. Ten-fold standard serial dilutions (10^7^ to 10^3^ molecules/μl) were run on each 96-well plate, with amplification efficiencies ranging from 1.95 to 2.00 and R^2^ = 0.99 to 1.00.

### Microbial function prediction

Microbial function prediction for each ileal, cecal and fecal sample based on 16S rRNA gene sequencing data was determined using Pylogenetic Investigation of Communities by Reconstruction of Unobserved States (PICRUSt) according to RFI rank, geographical location and the intestinal site [27]. Closed-reference OTU picking was performed at 97% similarity level against the Greengenes database (downloaded from http://greengenes.secondgenome.com/downloads/database/13_5), and processed in the online Galaxy PICRUSt interface (http://galaxyproject.org/) with a workflow described by the developers (http://picrust.github.com/picrust/tutorials/quickstart.html#quickstartguide). Sequences were categorized by function based on Kyoto Encyclopedia of Genes and Genomes (KEGG) pathways in PICRUSt. Non-bacteria related KEGG orthology functions and functions <0.01% relative abundance were dismissed.

### Statistical analyses

Sequencing data were included in the statistical analysis that were detected in more than 50% of the chickens, separate per location, gut site and sex. Feed efficiency and microbiota variables were first analyzed for normality using Shapiro-Wilk test with the PROC UNIVARIATE method in SAS, version 9.4. The Cook’s distance (Cook’s D) test was used to determine any influential observation on the model. Absolute 16S rRNA gene copies and sequencing data including α-diversity indices and relative abundances of individual phyla, families, genera and OTUs were analyzed by ANOVA using two different models and the MIXED procedure in SAS. The first model accounted for the fixed effects of sex, intestinal site, batch, geographical location and RFI group. Because chickens were sacrificed at different days of life and to consider that chickens were consecutively sampled, the first model included the random effects of chicken nested within day of life and chicken order at sacrifice. Sex as fixed effect was significant for most parameters; therefore, variables were analyzed separately for female and male chickens using the second model. This model was fitted to take into account the fixed effect of RFI group and geographical location and their two-way-interaction, separately per intestinal site. As previously reported in the literature [7,9], batch affected many bacterial taxa. Therefore, the random effect considered the chicken nested within batch, day of life and chicken order at slaughter. Degrees of freedom were approximated by the method of Kenward-Roger. The rigorous statistical tests applied in SAS allow a False Discovery Rate of less than 5%. Least squares means were computed using the pdiff statement and significance was declared at *P* ≤ 0.05. A trend was considered at 0.05 < *P* ≤ 0.10.

To characterize relationships between FE and performance traits and bacterial taxa and predicted metabolic functions that were similarly directed at both locations and to distinguish whether relationships were more predominant with bacterial taxa of high or low abundance, Pearson’s correlation analysis (CORR procedure of SAS) was used to identify individual genera, OTUs and KEGG pathways that were associated with RFI, TFI TBWG and FCR across locations, separately for female and male chickens, and to quantify their relationships in ileal and cecal digesta and feces. Moreover, Pearson’s correlations between KEGG pathways and bacterial genera that were associated with RFI were calculated to relate RFI-associated differences in the predicted metabolic functions back to bacterial abundances. Correlations between OTUs and RFI, FCR and performance traits were only calculated for individual OTUs, which occurred at a relative abundance >0.01% of all sequences at each intestinal site, sex and geographical location. Descriptive statistics was performed using the MEANS procedure of SAS (SAS Inst. Inc., version 9.4). In the following, the term cross-locational association will be used to describe similar effects across the two geographical locations.

## Results

### Feed efficiency

The RFI values and TFI of chickens within the same RFI rank were similar between geographical locations (Table 1). The RFI increased (*P* < 0.001) by 292 and 499 g from low (good FE) to high RFI (poor FE) in females and by 486 and 442 g from low to high RFI in males from L1 and L2, respectively (Table 1). By contrast, TBWG was equal for low and high RFI ranks, but it was lower in chickens from L2 who gained about 350 to 400 g less compared to chickens from L1 (*P* < 0.001). Chicken’s FCR values corresponded to those of the RFI values in low and high RFI groups. However, the location effect showed that chickens from L2 had higher FCR values and thus poorer FE than chickens from L1 (*P* < 0.001).

**Table 1:** Performance and feed efficiency data of low and high residual feed intake (RFI) broiler chickens raised at two geographical locations (L).

	L1^a^		L2^a^			*P-*Value		
Item	Low RFI	High RFI	Low RFI	High RFI	SEM	RFI	L	RFI × L
Females								
TFI, d 7–36 of life (g)	3334	3751	3559	3797	127.1	0.027	0.479	0.566
TBWG, d 7–36 of life (g)	2251	2279	1966	1856	69.9	0.647	<0.001	0.643
RFI (g)	-195	97	-267	232	28.4	<0.001	0.412	0.201
FCR (g/g)	1.46	1.62	1.65	1.89	0.027	<0.001	<0.001	0.108
Males								
TFI, d 7–36 of life (g)	3682	4185	3823	4321	99.1	<0.001	0.340	0.573
TBWG, d 7–36 of life (g)	2573	2615	2228	2214	77.1	0.582	<0.001	0.560
RFI (g)	-183	303	-211	231	30.9	<0.001	0.149	0.610
FCR (g/g)	1.41	1.61	1.58	1.79	0.028	<0.001	<0.001	0.774

Data are presented as least-square means and pooled standard error of the mean (SEM).

^a^ L1, University of Veterinary Medicine Vienna (Vienna, Austria); L2, Agri-Food and Biosciences Institute (Hillsborough, Northern Ireland, UK). TFI, total feed intake; TBWG, total body weight gain; FCR, feed conversion ratio.

### Absolute 16S rRNA gene abundance

Total bacterial 16S rRNA gene copy numbers in intestinal and fecal samples were affected by intestinal site, geographical location and RFI (Table 2). Cecal digesta comprised 1.5 and 1 log units more bacterial 16S rRNA log_10_ gene copies compared to ileal digesta and feces, respectively (*P* < 0.001, Table 2). In addition, females and males at L1 contained 1.1 and 1.3 log units more gene copies in the ileum and 0.2 and 0.4 log units more in the ceca, respectively, compared to L2 (*P* < 0.05), whereas 16S rRNA log_10_ gene copies in feces were similar. Small, but physiologically not relevant, RFI-related differences in the total bacterial gene copies were observed in the ceca of males (*P* < 0.05) and feces of females (*P* < 0.10), with low RFI chickens having 0.2 and 0.4 log units more gene copies compared to high RFI animals, respectively.

**Table 2:** Total bacterial gene copy numbers (log_10_ 16S rRNA gene copies/g fresh matter) in intestinal digesta and feces of low and high residual feed intake (RFI) broiler chickens raised at two geographical locations (L).

	L1^a^		L2^a^			*P*-Value^b^		
Item	Low RFI	High RFI	Low RFI	High RFI	SEM	RFI	L	RFI × L
Females								
Ileum	10.1	9.9	8.7	9.2	0.21	0.581	<0.001	0.177
Ceca	11.2	11.2	11.1	10.9	0.09	0.153	0.012	0.497
Feces	10.3	9.9	10.0	9.7	0.17	0.068	0.214	0.985
Males								
Ileum	10.2	10.3	8.8	9.1	0.23	0.440	<0.001	0.726
Ceca	11.3	11.0	10.8	10.7	0.07	0.012	<0.001	0.460
Feces	10.3	10.2	10.0	10.1	0.13	0.659	0.124	0.405

Data are presented as least-square means and pooled standard error of the mean (SEM).

^a^ L1, University of Veterinary Medicine Vienna (Vienna, Austria); L2, Agri-Food and Biosciences Institute (Hillsborough, Northern Ireland, UK).

^b^ Intestinal site affected total bacterial gene copy numbers (*P* < 0.001).

### Bacterial community composition related to intestinal site

From the 8,600,328 stitched reads for the 68 ileal, 68 cecal and 69 fecal samples (mean Phred score of 29 to 36) obtained from the commercial provider, 7,726,361 reads remained after quality control, with a mean of 37,690 reads per sample (min: 145, max: 175,973) and a mean read length of 557 ± 16 bp, which were classified into 6,404 OTUs.

**Fig 1:**
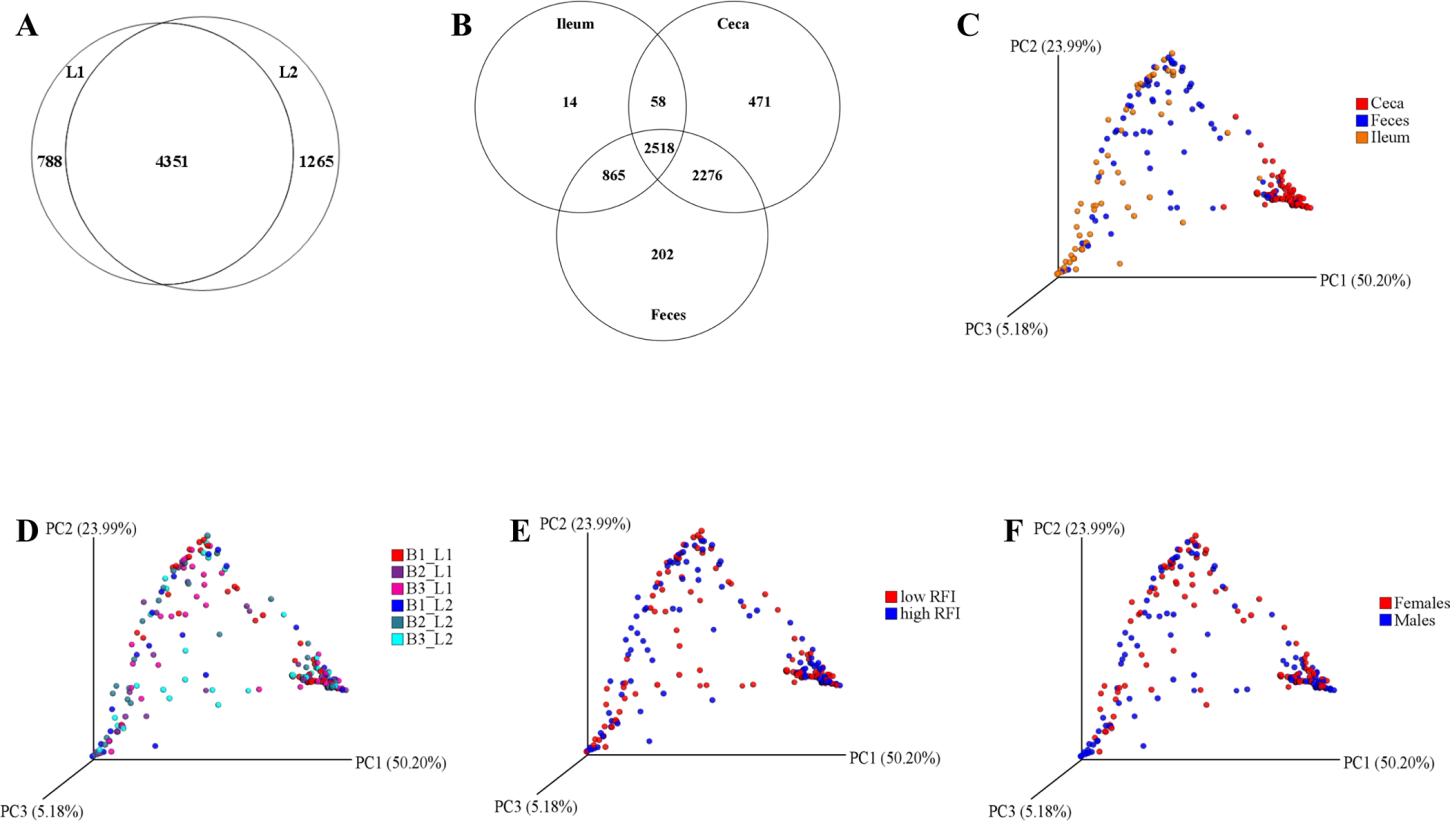
Venn diagrams and β-diversity plot. (A) Venn diagram showing the number of shared operational taxonomic units between two geographical locations (L1 and L2); (B) Venn diagram showing the number of shared operational taxonomic units between ileum, ceca and feces; principal coordinate analysis plots of weighted UniFrac analysis colored by (C) intestinal site; D) batch and geographical location; E) RFI ranks; and F) sex. Rarefaction depth of 10,000 sequences per sample removed 9 samples from the dataset; for ileum, n = 31 low RFI chickens and n = 30 high RFI chickens; for ceca, n = 32 low RFI chickens and n = 35 high RFI chickens; for feces, n = 33 low RFI chickens and n = 35 high RFI chickens.

**Fig 2:**
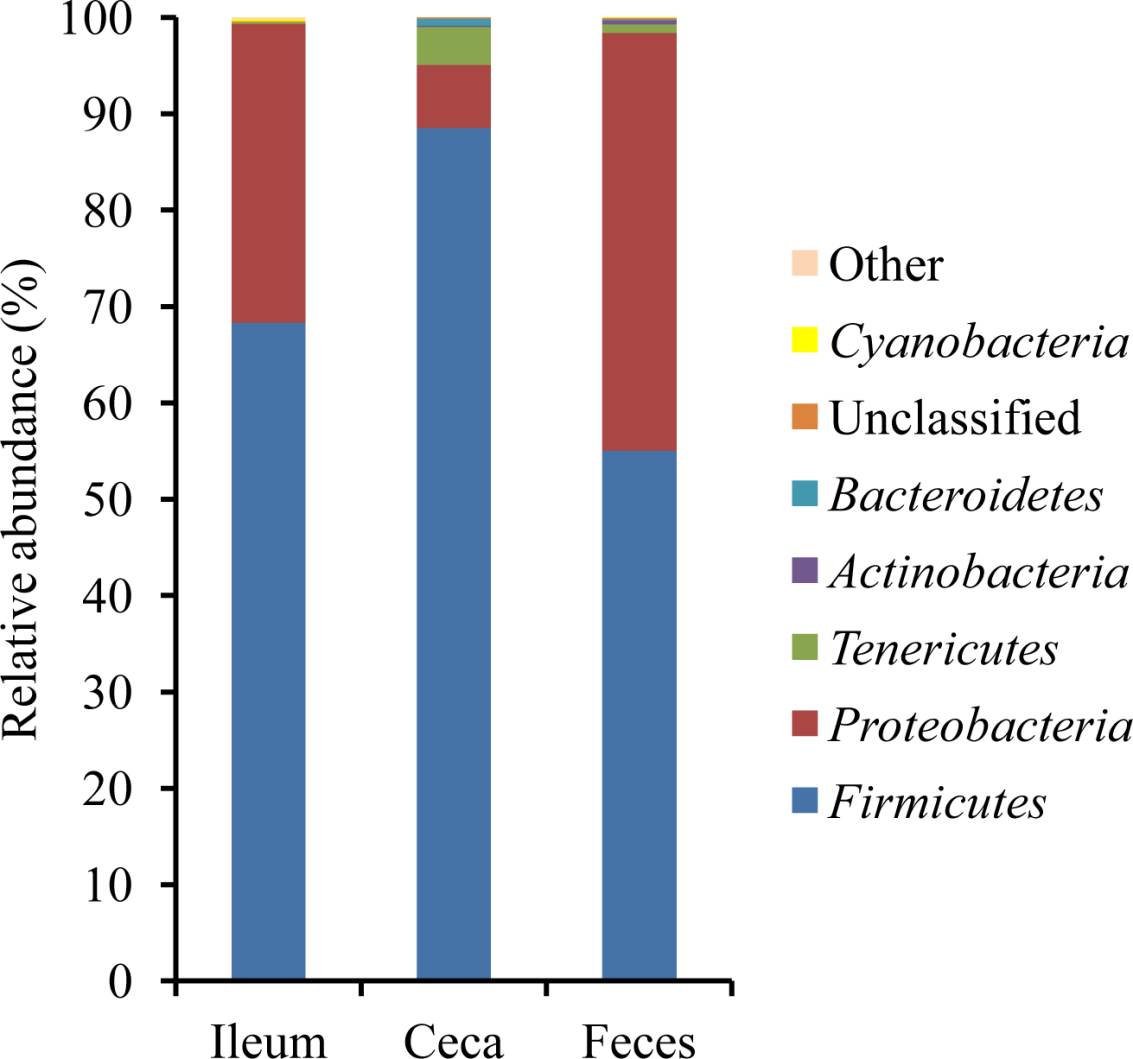
Relative abundance (%) of the most abundant phyla in ileal and cecal digesta and feces of broiler chickens raised at two geographical locations.

For all intestinal sites, the Venn diagram (Fig 1A) showed that 67.9% of all OTUs (4,351 OTUs) were shared among both geographical locations, whereas 12.3% and 19.8% of the OTUs were specifically associated with L1 and L2, respectively. Thereby, the shared OTUs represented 97.8% of all reads and comprised the high-abundance OTUs, whereas only low-abundance OTUs were specific for the geographical location. Moreover, 39.3% of all OTUs (2,518 OTUs) were shared among all three intestinal sites, whereby they corresponded to 98.1% of all reads (Fig 1B). In addition, more OTUs were shared between ceca and feces (74.9%) than between ileum and feces (52.8%). Despite the shared OTUs, β-diversity analysis using weighted UniFrac showed a clear separation of the community in cecal digesta from those in feces and ileal digesta (Fig 1C), which was confirmed by intestinal-site specific relative abundances at phylum and genus level (*P* < 0.05; Fig 2; S2 Table). This was also visible when comparing the taxonomical composition. Particularly, bacteria belonging to the phylum *Firmicutes* were 20 to 35% more abundant in cecal digesta compared to ileal digesta and feces, respectively, whereas in ileal digesta and feces more *Proteobacteria* were present (*P* < 0.05; Fig 2). Moreover, the ileal community was predominated by the genera *Lactobacillus* (31.2%), *Escherichia/Hafnia/Shigella* (26.4%) and *Turicibacter* (24.0%; S2 Table). In cecal digesta, an unclassified *Clostridiales* genus (55.7%) and an unclassified *Ruminococcaceae* (15.8%) predominated, whereas in feces an *Escherichia/Hafnia/Shigella* (39.1%), *Turicibacter* (14.0%) and an unclassified *Clostridiales* genus (13.8%) were highly abundant.

### Changes in bacterial community related to geographical location

Beta-diversity analysis using weighted UniFrac did not show specific clustering based on the different batches and the different geographical locations (Fig 1D). Nevertheless, effects of the geographical location were detectable when comparing species richness and α-diversity (Table 3) as well as the relative abundance of several phyla (Table 4) and genera (Tables 5 and 6) across the two locations. In males from L1, the cecal community had a lower α-diversity as indicated by the Shannon and Simpson indices (*P* < 0.05) compared to males at L2 (Table 3). For the fecal community in females, in contrast, the Chao1 (*P* < 0.05) and Shannon (*P* < 0.10) indices showed higher species richness and diversity in animals at L1 compared to L2.

**Table 3:** Differences in α-diversity indices in intestinal digesta and feces of low and high residual feed intake (RFI) broiler chickens raised at two geographical locations (L).

	L1^c^		L2^c^			*P*-Value^d^^,^^e^		
Item	Low RFI	High RFI	Low RFI	High RFI	SEM	RFI	L	RFI × L
Females								
Ileum								
Shannon	2.34	2.80	2.39	3.05	0.453	0.231	0.748	0.830
Simpson	0.53	0.64	0.59	0.71	0.089	0.220	0.475	0.998
Chao1	449	421	315	450	61.2	0.398	0.406	0.200
Ceca								
Shannon	5.28^b^	5.28^b^	4.78^b^	6.41^a^	0.299	0.012	0.311	0.012
Simpson	0.93^a^	0.92^a^	0.83^b^	0.96^a^	0.026	0.020	0.295	0.014
Chao1	1076^b^^A^	1084^b^^A^	807^b^^B^	1365^a^	96.0	0.007	0.951	0.008
Feces								
Shannon	3.60	3.21	2.78	2.44	0.413	0.389	0.065	0.954
Simpson	0.72	0.66	0.57	0.59	0.068	0.730	0.134	0.605
Chao1	772	594	525	437	87.8	0.142	0.030	0.613
Males								
Ileum								
Shannon	2.16	2.79	2.42	3.11	0.320	0.048	0.378	0.919
Simpson	0.55	0.67	0.62	0.69	0.061	0.128	0.447	0.708
Chao1	310	347	305	457	63.0	0.145	0.414	0.370
Ceca								
Shannon	5.49	5.39	6.21	6.13	0.205	0.664	0.001	0.974
Simpson	0.93	0.91	0.96	0.94	0.014	0.180	0.044	0.957
Chao1	1152	1050	1191	1260	90.0	0.856	0.174	0.348
Feces								
Shannon	3.83	3.65	4.13	3.38	0.420	0.280	0.966	0.504
Simpson	0.79	0.77	0.79	0.72	0.045	0.338	0.562	0.559
Chao1	729	526	801	633	97.5	0.066	0.365	0.858

Data are presented as least-square means and pooled standard error of the mean (SEM).

^a,b^ Different superscript letters within a row indicate significant difference (*P* ≤ 0.05).

^A,B^ Different superscript capital letters within a row indicate a tendency (*P* ≤ 0.10).

^c^ L1, University of Veterinary Medicine Vienna (Vienna, Austria); L2. Agri-Food and Biosciences Institute (Hillsborough, Northern Ireland, UK).

^d^ Rarefaction depth of 10,000 sequences per sample removed 9 samples from the dataset. L1, n = 9/sex, RFI and intestinal site, except for ileum where n = 8 high RFI females, and n = 8 low RFI males, and for feces where n = 8 low RFI females; L2, for ileum, n = 5 low RFI females, n = 5 high RFI females, and n = 9 low RFI males, n = 8 high RFI males; for ceca, n = 4 low RFI females, n = 8 high RFI females, and n = 10 low RFI males, n = 9 high RFI males; for feces, n = 6 low RFI females, n = 8 low RFI females, and n = 10 low RFI males, n = 9 high RFI males.

^e^ Intestinal site affected all diversity indices (*P* < 0.001).

**Table 4:** Differences in the relative abundance (%) of bacterial phyla in intestinal digesta and feces of low and high residual feed intake (RFI) broiler chickens raised at two geographical locations (L).

	L1^c^		L2^c^			*P*-Value^d^		
Phylum	Low RFI	High RFI	Low RFI	High RFI	SEM	RFI	L	RFI × L
Females								
Ileum								
*Firmicutes*	81.93^a^	53.50^b^	56.66^a^^b^	72.91^a^^b^	10.370	0.563	0.781	0.041
*Proteobacteria*	17.57^b^	46.28^a^	41.47^a^^b^	25.64^a^^b^	10.372	0.541	0.877	0.042
*Cyanobacteria*	0.004	0.02	0.95	0.42	0.366	0.485	0.079	0.460
*Actinobacteria*	0.01	0.03	0.74	0.37	0.277	0.541	0.063	0.497
Unclassified	0.10^A^	0.04	0.01^B^	0.08	0.032	0.901	0.466	0.066
Ceca								
*Firmicutes*	94.86	90.67	84.36	80.50	3.516	0.267	0.007	0.963
*Tenericutes*	1.60^b^	1.28^b^	2.25^b^	7.94^a^	0.911	0.007	<0.001	0.003
*Bacteroidetes*	0	0	1.16	1.63	0.354	0.519	<0.001	0.520
*Actinobacteria*	0	0	0.38	0.23	0.071	0.331	<0.001	0.332
Unclassified	0.11^a^^b^^B^	0.11^a^^b^^B^	0.05^b^	0.22^a^^A^	0.041	0.044	0.627	0.054
Feces								
Unclassified	0.34	0.26	0.10	0.18	0.083	0.999	0.075	0.337
*Actinobacteria*	0.008^b^	0.06^b^	0.23^a^^A^	0.06^a^^b^^B^	0.057	0.284	0.062	0.060
*Bacteroidetes*	0^b^	0^b^	0.08^a^	0.02^b^	0.012	0.011	<0.001	0.014
*Cyanobacteria*	0.008^b^	0.009^b^	0.07^a^	0.005^b^	0.018	0.092	0.122	0.084
Males								
Ileum								
*Cyanobacteria*	0.003	0.01	0.38	0.27	0.152	0.724	0.042	0.708
*Actinobacteria*	0.06	0.09	0.26	0.21	0.092	0.903	0.089	0.686
Ceca								
*Firmicutes*	93.10	95.13	85.42	86.73	1.870	0.379	<0.001	0.849
*Tenericutes*	1.10	2.42	7.34	6.09	1.043	0.972	<0.001	0.226
*Bacteroidetes*	0	0	1.44	1.26	0.430	0.837	0.004	0.838
*Actinobacteria*	0	0	0.37	0.26	0.056	0.361	<0.001	0.359
Unclassified	0.11	0.12	0.16	0.20	0.036	0.536	0.065	0.610
Feces								
*Tenericutes*	0.45	0.01	3.58	0.51	0.873	0.058	0.051	0.130
*Bacteroidetes*	0	0	0.23	0.10	0.071	0.478	0.042	0.271

Data are presented as least-square means and pooled standard error of the mean (SEM).

^a,b^ Different superscript letters within a row indicate significant difference (*P* ≤ 0.05).

^A,B^ Different superscript capital letters within a row indicate a tendency (*P* ≤ 0.10).

^c^ L1, University of Veterinary Medicine Vienna (Vienna, Austria); L2. Agri-Food and Biosciences Institute (Hillsborough, Northern Ireland, UK).

^d^ Intestinal site affected *Firmicutes*, *Proteobacteria*, *Tenericutes*, *Bacteroidetes*, unclassified phylum (*P* < 0.001), and *Cyanobacteria* (*P* < 0.05).

**Table 5:** Differences in the relative abundance (%) of bacterial genera in intestinal digesta and feces of low and high residual feed intake (RFI) female broiler chickens raised at two geographical locations (L).

	L1^d^		L2^d^			*P*-Value		
Genus^c^	Low RFI	High RFI	Low RFI	High RFI	SEM	RFI	L	RFI × L
Ileum								
*Turicibacter*	55.37^a^^A^	24.78^a^^b^^B^	9.32^b^	21.94^a^^b^^B^	12.281	0.472	0.058	0.091
*Escherichia/Hafnia/Shigella*	15.91^b^	44.98^a^	35.49^a^^b^	14.85^b^	9.923	0.675	0.601	0.019
*Lactobacillus*	12.05	4.68	35.29	39.11	11.225	0.876	0.016	0.623
*Streptococcus*	0.69^b^	8.05^a^	0.46^b^	0.30^b^	1.548	0.029	0.016	0.023
Unclassified *Clostridiaceae* 2	0.16	0.31	0.80	1.08	0.364	0.551	0.064	0.864
*Methylobacterium*	0.004	0.01	1.22	0.65	0.509	0.584	0.081	0.573
*Bacillus*	0.006	0.02	0.46	0.16	0.173	0.412	0.096	0.371
*Clostridium*	0	0	0.07	0.06	0.021	0.849	0.020	0.503
*Corynebacterium*	0	0	0.02	0.07	0.016	0.059	0.025	0.169
Ceca								
Unclassified *Ruminococcaceae*	21.46	19.20	18.73	10.59	3.238	0.123	0.094	0.377
*Anaerotruncus*	7.29	8.39	0.63	1.75	2.016	0.590	0.003	0.999
Unclassified *RF39*	1.60^b^	1.28^b^	2.25^b^	7.94^a^	0.911	0.007	<0.001	0.003
*Ruminococcus*	1.92	2.15	4.60	4.32	0.529	0.967	<0.001	0.633
Unclassified *Lachnospiraceae* 2	0.63	0.56	2.36	1.29	0.545	0.311	0.034	0.370
*Faecalibacterium*	0.21	0.18	0.84	1.82	0.339	0.180	0.003	0.152
*Bacillus*	0.47	0.61	0.08	0.39	0.176	0.216	0.096	0.636
*Streptococcus*	0.32	0.64	0	0	0.160	0.061	0.059	0.980
Unclassified *Clostridiaceae* 2	0.004	0.005	0.23	0.03	0.069	0.157	0.079	0.150
*Clostridium*	0.03	0.04	0.004	0.009	0.011	0.441	0.020	0.744
Feces								
*Turicibacter*	7.99	3.39	17.16	32.19	7.653	0.503	0.020	0.212
Unclassified *Clostridiales* 1	18.18	12.73	4.11	0.89	5.337	0.426	0.022	0.837
Unclassified *Ruminococcaceae*	9.65	9.28	1.88	0.23	4.229	0.814	0.058	0.882
*Streptococcus*	4.12	5.88	0.45	1.29	1.853	0.491	0.035	0.807
Unclassified *Clostridiales* 2	0.15^b^	0.67^b^	6.57^a^	0.77^b^	1.817	0.159	0.085	0.094
Unclassified *Clostridiaceae* 2	0.09	0.17	2.79	1.30	0.514	0.185	<0.001	0.139
*Ruminococcus*	1.51	1.48	0.74	0.07	0.483	0.480	0.033	0.510
*Acinetobacter*	0.02^b^	1.74^a^	0.15^b^	0.12^b^	0.511	0.111	0.158	0.099
*Oscillospira*	0.82	0.76	0.29	0.04	0.272	0.566	0.028	0.731
*Anaerotruncus*	0.44	0.76	0.05	0.01	0.304	0.653	0.074	0.557
Unclassified *Lachnospiraceae* 2	0.28	0.19	0.41	0.02	0.124	0.060	0.883	0.243
Unclassified *Peptostreptococcaceae*	0.002	0.005	0.06	0.15	0.050	0.374	0.060	0.401
*Pseudomonas*	0.005^b^	0.004^b^	0.02^a^	0.003^b^	0.003	0.037	0.091	0.042

Data are presented as least-square means and pooled standard error of the mean (SEM).

^a,b^ Different superscript letters within a row indicate significant difference (*P* ≤ 0.05).

^A,B^ Different superscript capital letters within a row indicate a tendency (*P* ≤ 0.10).

^c^ The coverage of analyzed bacterial genera accounted for 99.02% of all sequences.

^d^ L1, University of Veterinary Medicine Vienna (Vienna, Austria); L2. Agri-Food and Biosciences Institute (Hillsborough, Northern Ireland, UK).

**Table 6:** Differences in the relative abundance (%) of bacterial genera in intestinal digesta and feces of low and high residual feed intake (RFI) male broiler chickens raised at two geographical locations (L).

	L1^d^		L2^d^			*P*-Value		
Genus^c^	Low RFI	High RFI	Low RFI	High RFI	SEM	RFI	L	RFI × L
Ileum								
*Lactobacillus*	16.78^b^	48.70^a^^b^	57.13^a^	34.36^a^^b^	13.658	0.740	0.348	0.054
*Turicibacter*	37.96^a^	7.51^b^	6.22^b^	28.58^a^^b^	9.499	0.673	0.578	0.009
*Streptococcus*	3.88	5.04	0.08	0.71	1.835	0.631	0.034	0.886
*Klebsiella*	1.88^a^	0.19^b^	0.05^b^	0.57^a^^b^	0.559	0.302	0.206	0.056
*Methylobacterium*	0.006	0.003	0.40	0.39	0.225	0.976	0.091	0.985
*Pseudomonas*	0	0	0.08	0.06	0.036	0.713	0.091	0.844
Ceca								
Unclassified *Clostridiales* 1	45.62	59.38	60.66	63.48	3.878	0.040	0.019	0.168
Unclassified *Ruminococcaceae*	23.01^a^	15.99^b^^A^	9.66^b^^B^	10.59^b^	2.327	0.199	<0.001	0.097
*Anaerotruncus*	8.72	11.32	1.36	1.26	2.010	0.538	<0.001	0.506
Unclassified *RF39*	1.10	2.42	7.34	6.09	1.043	0.972	<0.001	0.226
*Ruminococcus*	2.83	1.47	4.28	3.57	0.460	0.031	<0.001	0.479
*Faecalibacterium*	0^c^	0^c^	2.29^a^	1.06^b^	0.313	0.120	<0.001	0.026
*Bacillus*	1.20	0.83	0.15	0.21	0.296	0.594	0.008	0.466
*Streptococcus*	1.05^a^	0.35^b^	0^b^	0^b^	0.165	0.024	<0.001	0.074
Unclassified *Christensenellaceae*	0.26	0.20	0.64	0.48	0.101	0.280	0.002	0.653
*Coprococcus*	0.24	0.16	0.17	0.12	0.032	0.050	0.131	0.626
Unclassified *Clostridiales* 2	0.24	0.17	0.11	0.12	0.031	0.358	0.006	0.190
*Clostridium*	0.03	0.04	0.006	0.03	0.009	0.142	0.076	0.361
Feces								
*Lactobacillus*	10.09	16.10	7.39	28.56	7.918	0.096	0.542	0.346
Unclassified *Ruminococcaceae*	8.53	5.45	2.55	1.88	2.563	0.471	0.072	0.642
*Acinetobacter*	0.51^b^	4.51^a^	0.12^b^	0.12^b^	0.955	0.044	0.018	0.044
Unclassified *RF39*	0.45	0.10	3.60	0.51	0.873	0.058	0.051	0.130
*Klebsiella*	2.10	2.18	0.04	0.05	1.089	0.967	0.063	0.973
Unclassified *Clostridiales* 2	0.19	0.09	1.30	1.91	0.509	0.618	0.007	0.488
Unclassified *Clostridiaceae* 2	0.20^b^	0.05^b^	0.16^b^	1.11^a^	0.272	0.154	0.070	0.050
*Faecalibacterium*	0	0	0.74	0.61	0.300	0.967	0.046	0.704
*Anaerotruncus*	0.57	0.53	0.12	0.08	0.193	0.844	0.025	0.993
Unclassified *Lachnospiraceae* 1	0.42	0.12	0.41	0.23	0.131	0.078	0.700	0.668
*Blautia*	0.04	0.02	0.14	0.16	0.067	0.994	0.087	0.747
*Dorea*	0.04	0.02	0.07	0.02	0.017	0.026	0.277	0.527
Unclassified *Peptostreptococcaceae*	0	0	0.01	0.03	0.006	0.493	0.009	0.169

Data are presented as least-square means and pooled standard error of the mean (SEM).

^a,b^ Different superscript letters within a row indicate significant difference (P ≤ 0.05).

^A,B^ Different superscript capital letters within a row indicate a tendency (P ≤ 0.10).

^c^ The coverage of analyzed bacterial genera accounted for 99.02% of all sequences.

^d^ L1, University of Veterinary Medicine Vienna (Vienna, Austria); L2. Agri-Food and Biosciences Institute (Hillsborough, Northern Ireland, UK).

The phyla with the most pronounced differences for geographical location included *Tenericutes* and *Actinobacteria* in the ceca of both sexes and *Firmicutes* in cecal digesta of males (*P* < 0.001; Table 4). At genus level, strong effects for geographical location were found for the genera *Ruminococcus* in cecal digesta and an unclassified *Clostridiaceae 2* in the feces of females (*P* < 0.001; Table 5) as well as for unclassified *Ruminococcaceae*, *Anaerotruncus*, *Ruminococcus*, *Faecalibacterium* and *Streptococcus* in cecal digesta of males (*P* < 0.001; Table 6).

### Changes in the bacterial community related to RFI

Although the β-diversity analysis did not show RFI-associated clustering (Fig1E), comparison of the microbiota composition between RFI ranks demonstrated that RFI-related differences in bacterial abundances were evident, whereby RFI × location interactions suggested that many RFI effects were specific for the geographical location (Tables 4–6). Although sex-related clustering of intestinal samples was not observed when using weighted UniFrac analysis (Fig 1F), RFI effects and RFI × location interactions differed between sexes.

Specifically, the ceca of low RFI females comprised lower species richness (Chao1) and evenness (Shannon and Simpson) compared to high RFI females, but only at L2 but not at L1 (*P* < 0.05; Table 3). Low RFI males contained an ileal microbiota of lower evenness as indicated by the Shannon index (*P* < 0.05) and a fecal community of greater species richness (Chao1, *P* < 0.10) compared to high RFI males at both geographical locations.

Differences in the bacterial abundances that were associated with low and high RFI chickens were detectable at the phylum (Table 4) and genus level but differed for males and females (Tables 5 and 6). In females, a trend for cross-locational association with low RFI was found for a low-abundance unclassified *Lachnospiraceae* genus in feces (*P* < 0.1; Table 5). In contrast, in males, taxa that were enriched in low RFI animals across geographical locations were *Ruminococcus* (3.1% relative abundance) and the *Lachnospiraceae* genus *Coprococcus* (0.2% relative abundance) in cecal digesta, and, also belonging to the *Lachnospiraceae* family, *Dorea* in feces (<0.05% relative abundance; *P* < 0.05; Table 6). Cross-locational association with high RFI was observed for the most abundant genus, an unclassified *Clostridiales* genus, which was less abundant with low RFI compared to high RFI in cecal digesta of males (*P* < 0.05; Table 6).

Changes in bacterial genera that were associated with low RFI only at L1 included the high-abundance genus *Turicibacter* in ileal digesta of females (*P* < 0.10; Table 5) and males (*P* < 0.05; Table 6). Also in both sexes at L1, feces contained less *Acinetobacter* phylotypes with low RFI compared to high RFI (*P* < 0.05; Tables 5 and 6). In addition, in females, high- abundance *Escherichia*/*Hafnia*/*Shigella* and *Streptococcus* were less abundant with low RFI in ileal digesta (*P* < 0.05; Table 5), whereas in males, the cecal community comprised more *Streptococcus* with low RFI compared to high RFI at L1 (*P* < 0.05; Table 6).

Genera associated with low RFI at L2 differed from those at L1 and were an unclassified *Clostridiales* genus and *Pseudomonas* in feces of female chickens (*P* < 0.05; Table 5). Moreover, in females, high RFI was associated with the abundance of an unclassified *RF39* genus in cecal digesta (*P* < 0.05; Table 5). In males, in turn, low-abundance *Faecalibacterium* in cecal digesta and an unclassified *Clostridiaceae* genus in feces were enriched with low RFI (*P* < 0.05; Table 6).

### Relationships among bacteria and RFI and performance across geographical locations

Correlation analysis was performed between bacterial taxa at genus and species level, and the FE and performance variables (RFI, TFI, TBWG and FCR; Table 7, S3 and S4 Tables). From the bacteria that could be taxonomically assigned at family level, most relationships were with members of *Turicibacteraceae*, *Lactobacillaceae*, *Enterococcaceae*, *Ruminococcaceae*, *Lachnospiraceae* and several families from the *Proteobacteria* phylum. Correlation analysis confirmed our results for common variation in RFI-associated bacterial genera across geographical locations, whereby more correlations with high-abundance and low-abundance genera and species were found for TFI, TBWG and FCR than for RFI across all intestinal sites and both sexes (Table 7, S3 and S4 Tables). There were 5, 11, 11 and 12 significant correlations between bacterial genera and chicken’s RFI, TFI, TBWG and FCR (*P* < 0.05), respectively, irrespective of the sex. At species level, we found 12 positive and 12 negative correlations between RFI and OTUs across all intestinal sites and both sexes (S3 and S4 Tables). However, most correlations were with OTUs that showed only low similarity to their closest type strain by using the Greengenes 16S rRNA gene database (http://greengenes.lbl.gov/). Correspondingly, only eight OTUs could be taxonomically assigned to their closest type strain with >95% similarity. Those were, in females, one *Enterobacter*-OTU (OTU212, relative abundance <0.5%) in ileal digesta that correlated negatively with chicken’s RFI and two *Lactobacillus crispatus*-OTUs (OTU4 and OTU8, relative abundance >0.5%) in feces that correlated positively with chicken’s RFI (*P* < 0.05). In males, two *Eubacterium*-OTUs (OTU84 and OTU574) and one *Clostridium leptum*-OTU (OTU281) in cecal digesta correlated positively with chicken’s RFI, whereas one *Clostridium hylemonae-*OTU (OTU215) and one *Turicibacter*-OTU (OTU288) in feces correlated negatively with RFI (<0.5% relative abundance; *P* < 0.05). By contrast, 30 positive and 63 negative correlations between FCR and OTUs were observable across sexes and intestinal sites (S3 and S4 Tables).

**Table 7:** Selected genera correlating to feed efficiency and performance traits in low and high residual feed intake (RFI) female and male chickens (*P* < 0.05) across geographical locations and by intestinal site.

Genus^a^	RFI	TFI	TBWG	FCR	n	Mean	SE	Lower 95% CI	Upper 95% CI	5th Pctl	95th Pctl
Females											
Ileum											
*Turicibacter*	ns	-0.53	ns	-0.40	31	30.02	6.55	16.64	43.40	0.03	97.15
*Escherichia/Hafnia/Shigella*	ns	0.37	0.36	ns	31	27.90	5.25	17.18	38.62	0.06	83.90
*Streptococcus*	ns	ns	0.40	ns	31	2.70	0.96	0.74	4.65	0	14.42
*Enterococcus*	ns	0.41	ns	ns	31	0.15	0.06	0.02	0.28	0	1.12
Ceca											
Unclassified *Ruminococcaceae*	ns	ns	ns	-0.42	31	17.56	1.69	14.10	21.01	6.52	37.33
*Anaerotruncus*	ns	ns	0.41	ns	31	5.11	1.11	2.84	7.37	0.22	20.93
Unclassified *RF39*	ns	ns	ns	0.56	31	3.25	0.66	1.90	4.60	0.03	9.81
*Ruminococcus*	ns	ns	ns	0.60	31	3.04	0.33	2.37	3.71	1.02	6.78
Unclassified *Lachnospiraceae* 1	ns	0.38	ns	ns	31	1.72	0.37	0.96	2.49	0.08	8.64
*Faecalibacterium*	ns	ns	ns	0.49	31	0.72	0.20	0.31	1.13	0	2.77
*Dorea*	ns	0.38	ns	ns	31	0.25	0.05	0.16	0.35	0.009	0.97
Feces											
Unclassified *Clostridiales* 1	ns	ns	ns	-0.40	32	9.69	2.80	3.97	15.40	0.14	55.18
Unclassified *Ruminococcaceae*	ns	ns	ns	ns	32	5.73	2.13	1.38	10.08	0.03	26.07
*Lactobacillus*	0.36	ns	ns	0.47	32	4.94	1.61	1.66	8.23	0.009	27.70
Unclassified *Clostridiales* 2	-0.33	ns	-0.05	ns	32	1.65	0.96	-0.30	3.60	0.006	8.53
*Ruminococcus*	ns	ns	0.33	-0.36	32	1.00	0.25	0.49	1.51	0.01	4.57
Unclassified *Clostridiaceae* 1	ns	ns	-0.35	ns	32	0.77	0.54	-0.33	1.87	0	4.16
Males											
Ileum											
*Enterococcus*	ns	-0.33	-0.36	ns	37	0.72	0.37	-0.04	1.48	0	8.68
Unclassified *Clostridiaceae* 2	ns	ns	ns	0.34	37	0.37	0.12	0.13	0.61	0	2.52
Ceca											
Unclassified *Clostridiales*1	ns	ns	ns	0.36	37	57.38	2.18	52.96	61.79	31.96	79.33
*Ruminococcus*	-0.34	ns	ns	ns	37	3.07	0.28	2.50	3.64	0.82	6.37
*Coprococcus*	ns	ns	ns	-0.36	37	0.17	0.02	0.14	0.21	0.05	0.39
Feces											
Unclassified *Clostridiales* 1	ns	-0.33	ns	ns	37	17.66	3.95	9.65	25.67	0.08	67.48
*Lactobacillus*	ns	0.33	ns	ns	37	15.31	4.02	7.15	23.48	0.006	95.03
*Acinetobacter*	0.34	0.34	0.45	ns	37	1.28	0.55	0.17	2.40	0	11.86
*Proteus*	ns	ns	-0.41	0.35	37	0.52	0.39	-0.26	1.30	0	2.02
*Pseudomonas*	ns	0.36	0.34	ns	37	0.46	0.34	-0.23	1.15	0	4.78
*Enterococcus*	ns	-0.32	-0.35	ns	37	0.32	0.13	0.06	0.59	0	2.57
*Dorea*	-0.41	ns	ns	ns	37	0.04	0.01	0.02	0.06	0	0.20

^a^ ns, not significant; RFI, residual feed intake; TFI, total feed intake; TBWG, total body weight gain; FCR, feed conversion ratio; SE, standard error; CI, confidence interval; Pctl, percentile.

### Predicted microbial functions related to FE

**Fig 3:**
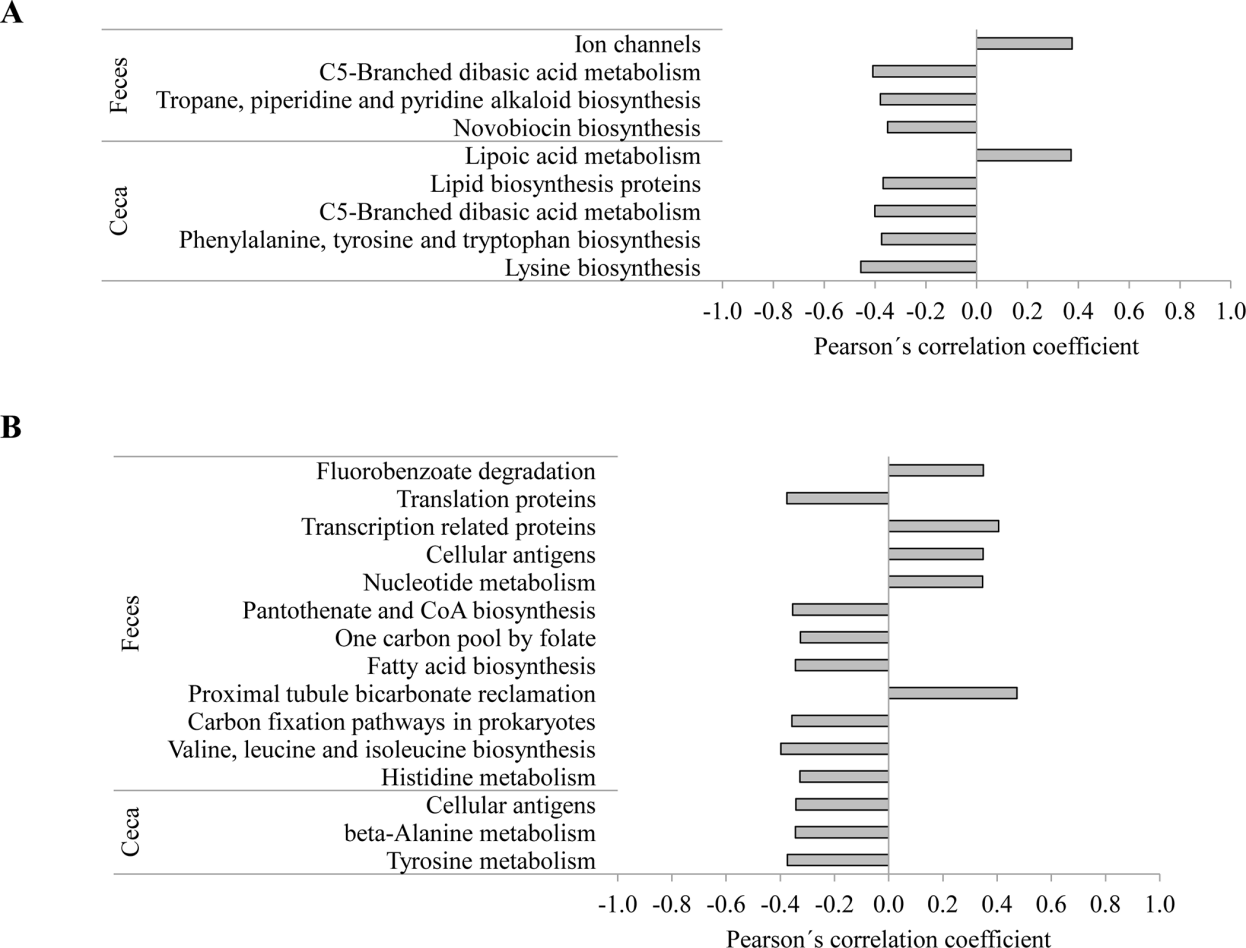
Selected KEGG pathways correlating to residual feed intake (RFI) in chickens across two geographical locations and by intestinal site; A) females and B) males.

In order to identify whether relationships between bacterial taxa and FE would be reflected in FE-associated variation in metabolic functions, predicted metagenome functionality of the ileal, cecal and fecal microbiota was inferred using the PICRUSt package and the KEGG database. Because our focus was on cross-locational FE- and performance-associated variation in predicted metabolic functions, data were only analyzed by correlation analysis. Across sexes, 24 significant correlations between RFI and KEGG pathway abundances existed mainly in cecal digesta and feces, particularly in feces of males, whereas they were absent in ileal digesta of both sexes (Fig 3A and 3B; S5 and S6 Tables). Those correlated pathways included amino acid, fatty acid and vitamin metabolism, cellular signaling and interaction and cell metabolism. However, 86 relationships between FCR and KEGG pathways could be established especially in cecal digesta and fewer in ileal digesta and feces of both sexes (S5 and S6 Tables). In cecal digesta, the FCR correlated with pathways especially in relation to amino acid, carbohydrate and lipid metabolism, vitamin synthesis as well as to the bacterial cell metabolism. Moreover, TFI and TBWG showed positive and negative correlations to amino acid, carbohydrate, vitamin and xenobiotics metabolism mainly in ileal digesta of females, whereas in males most correlations were found in feces (S5 and S6 Tables).

When correlating KEGG pathways with bacterial genera abundances that were both associated with RFI, one positive relation in cecal digesta of males (S8 Table) as well as in feces 21 negative and 24 positive associations in males (S9 Table) and six negative and three positive associations in females (S10 Table) were found across geographical locations.

## Discussion

Previous studies demonstrated associations between chicken’s intestinal microbiota composition and FE, but similar inter- and intra-study FE-associated bacterial profiles were hardly discernible [5,7,8]. Here, the ileal, cecal and fecal bacterial profiles of chickens ranked on RFI from two geographical locations were characterized together in order to expand our knowledge on RFI-associated microbiota profiles as targets for nutritional intervention. The latter can be only successful if bacteria associated with good FE (low RFI or FCR) are identifiable for multiple environments. Despite using different hatcheries, six different batches and preparing our diets locally, the intestinal microbiota was mostly similar at both geographical locations and only differed in very-low abundance bacteria in the present study. This finding differed from previous observations of different underlying microbiota when chickens were raised in different environments [7,9,10]. Nevertheless, differences in the bacterial structure and abundances at six weeks of age existed, indicating that the geographical location had modified the actual bacterial abundances.

RFI-associated variation in the ileal, cecal and fecal microbiota and predicted metabolic functions was detectable at both geographical locations. However, cross-locational relationships with chicken’s RFI could be mainly established with low-abundance OTUs. In contrast to hens where relationships between RFI and bacteria emphasized an important role of the cecum for chicken’s FE [8], we found more cross-locational RFI-relationships between RFI and OTUs and predicted metabolic functions in feces than in ileal or cecal digesta. The ceca are the site of the greatest intestinal fermentation in chickens [28] and more efficient fermentation may yield more energy for the host [29]. Due to the short transit time in the colon, the identified bacterial FE-associations were therefore likely less important for host nutrient and energy assimilation and the intestinal immune response compared to the FE-assocations found in ileal or cecal digesta. The fecal microbiota composition is mainly determined by the periodic emptying of the microbiota from the ileum and ceca [15]. Therefore, the intestinal origin is a main determinant for the chicken fecal microbiota composition and likely explains the absence of clear clustering of fecal samples from ileal and cecal samples in the PCoA plot [15]. Nevertheless, our results also support that the cecal and fecal RFI-associated microbiota cannot be applied to the bacterial community in the upper digestive tract of chickens [8]. Likewise, RFI-associated bacteria were different in males and females, indicating that FE-associated bacterial profiles cannot be transferred from one sex to the other.

Irrespective of the sex, more cross-locational associations between bacteria of low and high abundance and TFI, TBWG and FCR were observed in the present study, supporting interactions between the bacterial community and the host in the small and large intestines. Accordingly, negative relationships between FCR and predicted functions for amino acid, carbon and fatty acid metabolism may indicate a role of the ileal microbiota in nutrient acquisition for the host. As simple ratio trait, the FCR accounted for the slower growth of chickens from L2 compared to chickens from L1, which was apparent throughout all replicate batches and for both sexes. As per definition, the RFI was independent of the differences in growth performance between the two geographical locations. This led to our observation that low RFI chickens from L2 had a comparable FCR value as high RFI chickens from L1. Therefore, our data illustrate the importance to carefully select the FE metric when aiming to characterize FE-related microbiota profiles. For the present chicken populations, the FCR may have better reflected cross-locational physiological and microbial differences than the RFI. Because different selection strategies for FE (e.g. FCR, AME or RFI) were previously applied [5,7,8,30], this may have contributed to the inter-study variation in FE-associated bacterial taxa. This assumption is supported by findings in cattle where correlations between efficiency parameters and bacterial genera abundances differed depending on the FE metric used (i.e., FCR vs. RFI) [31].

Most bacteria showing cross-locational RFI-associations belonged to the two predominating phyla *Firmicutes* and *Proteobacteria*, which is in general accordance with previous findings [7,8,32]. Nevertheless, one important bacterial phylum for energy-harvesting, the *Bacteroidetes*, was almost absent in the current chicken populations. Its fecal abundance has been previously linked to high FCR and high RFI phenotypes in chickens [5,8]. We detected high numbers of *Bacteroidetes* bacteria in intestinal digesta using the same DNA extraction procedure in the past [16,17]. Since also others reported a lack of *Bacteroides* bacteria in the intestine of chickens [33], the present observation may be due to the experimental surroundings and the missing contact with the maternal microbiota [9].

Simply due to their dominance, taxonomic groups of high abundance may have a greater impact on nutrient assimilation of the host and host physiology than single low-abundance phylotypes [34]. However, low-abundance bacteria should not be seen individually, but as an integral part of the bacterial community, which interacts with the host [8]. Evidence from other ecological habitats corroborates that low-abundance microorganisms can markedly shape the ecosystem which they inhabit [35]. Furthermore, the absolute bacterial abundance will affect chicken’s digestive efficiency [34]. For this reason, the greater abundance of bacteria in ileal digesta of chickens from L1 compared to chickens from L2 may have yielded more energy from fermentation for the host and may help explaining the cross-locational variation in growth performance. Though, RFI-associated differences in bacterial numbers in ileum, ceca and feces played a negligible role in the present study. When comparing the relative microbiota composition between RFI ranks, separately for females and males, only the *Lactobacillus* genus and two *Lactobacillus crispatus*-OTUs in feces were indicative for high RFI in females at both geographical locations. Moreover, the relative abundances of the two *Lactobacillus crispatus*-OTUs in cecal digesta and feces were also indicative for low TBWG and high FCR values, respectively, again only in females, thereby confirming previous findings [30,33]. *Lactobacillus* have been shown the ability to activate both innate defense mechanisms and adaptive immune responses in chicken’s intestine [29,36,37]. Despite these potential benefits for intestinal health, activation of the immune system is generally energetically costly to the host and may alter partitioning of energy and nutrients away from growth towards processes that support the immune system response, which, in turn, may decrease chicken’s FE [38]. This may be supported by the positive correlation between *Lactobacillus* and predicted cellular antigens in feces of male chickens. Moreover, despite the short transit time in chicken’s intestine, fermentation of dietary residuals by *Lactobacillus* in the distal intestinal regions may have reduced SCFA generation as indicated by the negative association between this genus and predicted carbon fixation and fatty acid biosynthesis pathways in feces.

By contrast, other high-abundance genera, such as *Turicibacter* and *Escherichia/Hafnia/Shigella*, in ileal digesta showed no or contrasting RFI-associations between the two geographical locations and might indicate that both genera may inhabit similar intestinal niches. Nevertheless, correlations between *Turicibacter* and *Escherichia/Hafnia/Shigella* and TFI suggested that these two genera probably affected the host physiology or the feeding behavior of the host. Previous findings were inconsistent on the relationship between *Escherichia/Shigella* and chicken’s growth performance. Some authors reported a negative association between ileal *Escherichia*/*Shigella* and nutrient digestibility and weight gain [39], whereas others found positive associations between *Escherichia*/*Shigella* in feces and chicken’s FCR [2,5]. This discrepancy may be linked to the actual strains colonizing chicken’s intestine. Accordingly, some strains of *Escherichia coli* may confer beneficial physiological activities by reducing intestinal inflammation, increasing host innate immune functions or protecting the intestinal barrier against pathogens [40], which may have consequences for TBWG. Due to their predominance in ileal digesta and despite the short transit time in chicken’s small intestine, *Escherichia/Hafnia/Shigella* probably contributed to SCFA production in the distal small intestine of our chickens, thereby affecting host physiology and feeding behavior [37]. Overall, the ileal abundance of *Turicibacter* was unusually high in the current chicken populations as opposed to previous studies showing that the ileal community is dominated by *Lactobacillus* spp. [41]. These inconsistent findings may be related to the specific housing conditions and the lack of maternal microbiota transfer post-hatch [9] in the present study.

In males, mainly RFI-associations with low-abundance bacterial taxa existed in the present study. Accordingly, *Ruminococcus* in cecal digesta and *Dorea* in feces were indicative for good FE. The genus *Ruminococcus* is known for its ability to degrade complex carbohydrates and thus may have contributed to an improved degradation of dietary fiber [42]. In addition, metabolic cross-feeding of fermentation metabolites (e.g., hydrogen, lactate and substrate degradation products) is a widespread feature of various *Clostridiales* bacteria [42] and may have indirectly promoted the abundance of the *Ruminococcus* and *Dorea*. Both genera produce butyrate, which is the preferred energy source of colonocytes and has anti-inflammatory properties [29]. Furthermore, in males, gram-negative *Acinetobacter* in feces was indicative for high RFI and thus for poor FE. Since gram-negative bacteria express cellular antigens, e.g. lipopolysaccharides, recognition of these antigens can lead to an enhanced innate immune activation with release of cytokines from mast cells [29]. Increased production of pro-inflammatory cytokines can impair the intestinal integrity and epithelial function and subsequently reduce animal’s FE [43, 44]. The RFI-associated predicted microbial functions agreed with the compositional relationships in feces of males indicating that genes related to carbohydrate, energy and amino acid metabolism were related to low RFI, whereas a high abundance of genes for bacterial signaling and interaction (i.e. cellular antigens) increased chicken’s RFI. When relating the RFI-associated predicted metabolic functions to RFI-associated bacterial taxa, those were mainly correlated with genera belonging to the family *Lachnospiraceae* in feces of males and female chickens and *Ruminococcus* in the ceca of males. Accordingly, those genera appeared to have promoted the FE of their host, by stimulating fatty acid, amino acid and vitamin synthesis. Although these RFI-associations were observed in feces, due to the short intestinal transit time in chickens it is more likely that these associations reflected the situation in chicken’s colon.

In cattle, a less diverse, but more specialized rumen microbiome was proposed to promote the energy acquisition of the host, thereby improving animal's FE [45]. Consistently, low RFI males and females had a less diverse microbiota in the ileum and ceca, respectively. By contrast, communities with higher species richness are thought to use limiting resources more efficiently [46] which may explain the current cross-locational association between increased species richness in feces and low RFI in males. These findings also coincide with the greater number of cross-locational relationships between chicken’s RFI and fecal bacteria and predicted metabolic functions. As a result of the lower feed intake, the decreased intestinal filling probably slowed down the intestinal passage, which may have promoted the establishment of a more species rich community in the colon. Concurrently, undigested fibrous feed particles may have been degraded more efficiently, thereby improving chicken’s FE. Previous studies also reported lower diversity and increased species richness for the intestinal and fecal microbiota, but the affected intestinal sites differed from the present study [7,8].

In conclusion, the present results showed that RFI-associated bacterial profiles could be identified across different geographical locations, whereby consortia of mostly low-abundance taxa in the ileum, ceca and feces may play a role for FE in chickens. Although more RFI-related taxa were found in chicken feces, those may be less important for host metabolism and immunity. Therefore, mainly bacterial FE-associations found in ileal and cecal digesta may serve as useful targets for dietary strategies. Different RFI-associated profiles in the microbiota between sexes further suggested that data from one sex could not reflect the RFI-associations of the other sex. Moreover, the low similarity of many OTUs to reference phylotypes especially in the ceca, however, often impeded the prediction of their specific role in energy homeostasis and interaction with the host. Present correlations also suggested that FCR may better mirror physiological and microbial FE-associated differences across geographical locations.

## Supporting information

S1 Table Dietary ingredients and chemical composition of diets (on as-fed basis). (DOCX)

S2 Table Differences in the relative abundance (%) of most abundant genera in ileal and cecal digesta and feces of broiler chickens raised at two geographical locations. (DOCX)

S3 Table Most abundant operational taxonomic units (OTU) correlating to feed efficiency and performance traits in female chickens across two geographical locations and by intestinal site. (DOCX)

S4 Table Most abundant operational taxonomic units (OTU) correlating to feed efficiency and performance traits in male chickens across two geographical locations and by intestinal site. (DOCX)

S5 Table Selected KEGG pathways correlating to feed efficiency and performance traits in female chickens across two geographical locations and by intestinal site. (DOCX)

S6 Table Selected KEGG pathways correlating to feed efficiency and performance traits in male chickens across two geographical locations and by intestinal site. (DOCX)

S7 Table BLAST search results for most abundant operational taxonomic units (OTU) correlated to feed efficiency and performance traits in intestinal digesta and feces of broiler chickens raised at two geographical locations. (DOCX)

S8 Table Pearson’s correlations between selected bacterial genera and KEGG pathways in cecal digesta associated with the residual feed intake in male and female chickens across two geographical locations. (DOCX)

S9 Table Pearson’s correlations between selected bacterial genera and KEGG pathways in feces associated with the residual feed intake in male chickens across two geographical locations. (DOCX)

S10 Table Pearson’s correlations between selected bacterial genera and KEGG pathways in feces associated with the residual feed intake in female chickens across two geographical locations. (DOCX)
