# Supplementary material for: Intestinal microbiota profiles associated with low and high residual feed intake in chickens across two geographical locations
Source: PLoS One. 2017 Nov 15;12(11):e0187766. doi: 10.1371/journal.pone.0187766 (PMC5687768; doi:10.1371/journal.pone.0187766)
Supplement: S1 Table — (DOCX) [file pone.0187766.s001.docx]

**S1 Table. Dietary ingredients and analyzed chemical composition of experimental diets at the two geographical different locations.**

| Item | Starter (day 1-10 of life) | Grower (day 11-21 of life) | Finisher (day 22-42 of life) |
| --- | --- | --- | --- |
| Ingredient, g/kg as-fed | |  |  |
| Corn | 612 | 660 | 679 |
| Soybean meal | 331 | 282 | 260 |
| Soybean oil | 17.5 | 20.6 | 27.7 |
| Limestone flour | 11.0 | 9.8 | 7.0 |
| Salt | 2.0 | 2.0 | 2.3 |
| Dicalcium phosphate | 16.1 | 15.0 | 13.4 |
| Premix^a-c^ | 11.0 | 11.0 | 10.0 |
| Total | 1000 | 1000 | 1000 |
| Analyzed chemical composition, g/kg DM, at L1^d^ | |  |  |
| Dry matter | 926 | 923 | 914 |
| Crude protein | 243 | 223 | 216 |
| Crude ash | 69 | 62 | 55 |
| Analyzed chemical composition, g/kg DM, at L2^d^ |  |  |  |
| Dry matter | 908 | 902 | 902 |
| Crude protein | 221 | 219 | 209 |
| Crude ash | 94 | 81 | 72 |

^a^ Provided per kilogram of starter diet: vitamin A as retinyl acetate, 13,000 IU; vitamin D_3_ as cholecalciferol, 5,000 IU; vitamin E as alpha-tocopherol-acetate, 80 IU; vitamin K, 3 mg; thiamin, 3 mg; riboflavin, 9 mg; pyridoxine, 4 mg; vitamin B_12,_ 20 µg; biotin, 0.15 mg; calcium pantothenate, 15 mg; nicotinic acid, 60 mg; folic acid, 2 mg; 500 mg choline chloride; methionine, 3,405 mg; threonine, 745 mg; lysine, 2,812 mg; I, 1 mg as calcium iodate; Se, 0.35 mg as sodium selenite; Fe, 40 mg as ferrous sulphate; Mo, 0.5 mg as sodium molybdate; Mn, 100 mg as manganous oxide; Cu, 15 mg as copper sulfate; Zn, 100 mg as zinc oxide.

^b^ Provided per kilogram of grower diet: vitamin A as retinyl acetate, 10,000 IU; vitamin D_3_ as cholecalciferol, 5,000 IU; vitamin E as alpha-tocopherol-acetate, 50 IU; vitamin K, 3 mg; thiamin, 2 mg; riboflavin, 8 mg; pyridoxine, 3 mg; vitamin B_12,_ 15 µg; biotin, 0.12 mg; calcium pantothenate, 12 mg; nicotinic acid, 50 mg; folic acid, 2 mg; 400 mg choline chloride; methionine, 3,018 mg; threonine, 726 mg; lysine, 2,831 mg; I, 1 mg as calcium iodate; Se, 0.35 mg as sodium selenite; Fe, 40 mg as ferrous sulphate; Mo, 0.5 mg as sodium molybdate; Mn, 100 mg as manganous oxide; Cu, 15 mg as copper sulfate; Zn, 100 mg as zinc oxide.

^c^ Provided per kilogram of finisher diet: vitamin A as retinyl acetate, 10,000 IU; vitamin D_3_ as cholecalciferol, 5,000 IU; vitamin E as alpha-tocopherol-acetate, 50 IU; vitamin K, 3 mg; thiamin, 2 mg; riboflavin, 6 mg; pyridoxine, 3 mg; vitamin B_12,_ 15 µg; biotin, 0.12 mg; calcium pantothenate, 10 mg; nicotinic acid, 50 mg; folic acid, 1 mg; 350 mg choline chloride; methionine, 2,514 mg; threonine, 361 mg; lysine, 1,779 mg; I, 1 mg as calcium iodate; Se, 0.35 mg as sodium selenite; Fe, 40 mg as ferrous sulphate; Mo, 0.5 mg as sodium molybdate; Mn, 100 mg as manganous oxide; Cu, 15 mg as copper sulfate; Zn, 100 mg as zinc oxide.

^d^ L1, University of Veterinary Medicine Vienna (Vienna, Austria); L2. Agri-Food and Biosciences Institute (Hillsborough, Northern Ireland, UK).
