# Supplementary material for: Intestinal microbiota profiles associated with low and high residual feed intake in chickens across two geographical locations
Source: PLoS One. 2017 Nov 15;12(11):e0187766. doi: 10.1371/journal.pone.0187766 (PMC5687768; doi:10.1371/journal.pone.0187766)
Supplement: S2 Table — (DOCX) [file pone.0187766.s002.docx]

S2 Table. Differences in the relative abundance (%) of most abundant genera in ileal and cecal digesta and feces of broiler chickens raised at two geographical locations.

| Genus | Relative abundance (%)^d^ | | | SEM | *P*-Value |
| --- | --- | --- | --- | --- | --- |
|  | Ileum | Ceca | Feces |  |  |
| Unclassified *Clostridiales*1 | 3.0^c^ | 55.7^a^ | 13.8^b^ | 1.82 | <0.001 |
| *Escherichia/Hafnia/Shigella* | 26.4^b^ | 6.4^c^ | 39.1^a^ | 2.95 | <0.001 |
| *Lactobacillus* | 31.16^a^ | 0.13^c^ | 10.15^b^ | 2.945 | <0.001 |
| *Turicibacter* | 24.04^a^ | 1.39^c^ | 14.01^b^ | 2.647 | <0.001 |
| Unclassified *Ruminococcaceae* | 1.43^c^ | 15.84^a^ | 5.02^b^ | 0.930 | <0.001 |
| *Streptococcus* | 2.39^a^ | 0.35^b^ | 3.20^a^ | 0.581 | 0.003 |
| *Anaerotruncus* | 0.07^b^ | 5.10^a^ | 0.31^b^ | 0.404 | <0.001 |
| Unclassified *RF39* | 0.20^c^ | 3.92^a^ | 0.88^b^ | 0.270 | <0.001 |
| Unclassified *Clostridiales* 2 | 3.23^a^ | 0.18^b^ | 1.32^b^ | 0.639 | 0.003 |
| *Ruminococcus* | 0.28^c^ | 3.13^a^ | 0.99^b^ | 0.143 | <0.001 |
| *Klebsiella* | 0.70^abB^ | 0.08^b^ | 1.59^aA^ | 0.384 | 0.013 |
| *Oscillospira* | 0.19^c^ | 1.49^a^ | 0.49^b^ | 0.083 | <0.001 |
| Unclassified *Lachnospiraceae* 1 | 0.12^b^ | 1.56^a^ | 0.32^b^ | 0.119 | <0.001 |
| *Staphylococcus* | 0^B^ | 0^B^ | 1.52^A^ | 0.584 | 0.115 |
| *Acinetobacter* | 0.52^ab^ | 0^b^ | 0.89^a^ | 0.249 | 0.035 |
| *Faecalibacterium* | 0.12^b^ | 0.90^a^ | 0.29^b^ | 0.097 | <0.001 |
| Unclassified *Lachnospiraceae* 2 | 0.06^bB^ | 0.90^a^ | 0.26^bA^ | 0.085 | <0.001 |
| Unclassified *Clostridiaceae* 1 | 0.23^ab^ | 0 ^b^ | 0.89^a^ | 0.291 | 0.075 |
| Unclassified *Clostridiaceae* 2 | 0.47^a^ | 0.08^b^ | 0.66^a^ | 0.108 | <0.001 |
| *Bacillus* | 0.20^b^ | 0.49^a^ | 0.09^b^ | 0.088 | 0.004 |
| *Enterococcus* | 0.46^a^ | 0^b^ | 0.22^ab^ | 0.125 | 0.018 |
| Unclassified *Christensenellaceae* | 0^b^ | 0.42^a^ | 0.05^b^ | 0.028 | <0.001 |
| *Proteus* | 0.04^B^ | 0^B^ | 0.33^A^ | 0.123 | 0.148 |
| *Corynebacterium* | 0.03 | 0 | 0.38 | 0.172 | 0.211 |
| *Blautia* | 0.02^bB^ | 0.26^a^ | 0.08^bA^ | 0.025 | <0.001 |
| *Pseudomonas* | 0.04 | 0 | 0.23 | 0.105 | 0.237 |
| *Methylobacterium* | 0.31^a^ | 0^b^ | 0.02^b^ | 0.074 | 0.004 |
| *Coprococcus* | 0^c^ | 0.17^a^ | 0.09^b^ | 0.018 | <0.001 |
| *Dorea* | 0^b^ | 0.22^a^ | 0.05^b^ | 0.016 | <0.001 |
| *Clostridium* | 0.13^A^ | 0.02^B^ | 0.11 | 0.052 | 0.130 |
| Unclassified *Peptostreptococcaceae* | 0.21^A^ | 0^B^ | 0.03 | 0.077 | 0.129 |

Data are presented as least-square means and pooled standard error of the mean (SEM).

For ileum and ceca, n = 31 females, and n = 37 males; for feces, n = 32 females, and n = 37 males.

^a,b,c^ Different superscript letters within the row indicate significant difference (*P* ≤ 0.05).

^A,B^ Different superscript capital letters within the row indicate a tendency (*P* ≤ 0.10).

^d^ The most abundant genera accounted for 99.02% of all sequences.
