# Supplementary material for: Intestinal microbiota profiles associated with low and high residual feed intake in chickens across two geographical locations
Source: PLoS One. 2017 Nov 15;12(11):e0187766. doi: 10.1371/journal.pone.0187766 (PMC5687768; doi:10.1371/journal.pone.0187766)
Supplement: S3 Table — (DOCX) [file pone.0187766.s003.docx]

S3 Table. Most abundant operational taxonomic units (OTU) correlating to feed efficiency and performance traits in female chickens across two geographical locations and by intestinal site.

| OTU^a-c^ | Taxonomy (Genus)^d,e^ | n | RFI | TFI | TBWG | FCR | Mean | SE | Lower 95% CI | Upper 95% CI | 5th Pctl | 95th Pctl |
| --- | --- | --- | --- | --- | --- | --- | --- | --- | --- | --- | --- | --- |
| Ileum |  |  |  |  |  |  |  |  |  |  |  |  |
| OTU1 | *Escherichia*/*Hafnia*/*Shigella* | 31 | ns | 0.36 | ns | ns | 23.90 | 4.56 | 14.58 | 33.22 | 0.05 | 75.43 |
| OTU2 | *Turicibacter* | 31 | ns | -0.53 | ns | -0.40 | 27.05 | 5.90 | 15.01 | 39.09 | 0.03 | 85.74 |
| OTU11 | *Streptococcus* | 31 | ns | ns | 0.42 | ns | 1.83 | 0.62 | 0.56 | 3.10 | 0 | 9.60 |
| OTU15 | *Escherichia*/*Hafnia*/*Shigella* | 31 | ns | ns | 0.36 | ns | 1.36 | 0.25 | 0.84 | 1.87 | 0 | 4.31 |
| OTU35 | *Turicibacter* | 31 | ns | -0.55 | ns | -0.38 | 0.85 | 0.18 | 0.48 | 1.23 | 0 | 2.76 |
| OTU40 | *Turicibacter* | 31 | ns | -0.52 | ns | -0.47 | 0.88 | 0.31 | 0.24 | 1.51 | 0 | 5.30 |
| OTU62 | *Streptococcus* | 31 | ns | ns | 0.42 | ns | 0.27 | 0.09 | 0.09 | 0.44 | 0 | 1.25 |
| OTU81 | *Enterobacter* | 31 | ns | 0.38 | ns | ns | 0.20 | 0.04 | 0.12 | 0.28 | 0 | 0.69 |
| OTU138 | *Escherichia*/*Hafnia*/*Shigella* | 31 | ns | ns | 0.38 | ns | 0.06 | 0.01 | 0.04 | 0.08 | 0 | 0.17 |
| OTU148 | *Escherichia*/*Hafnia*/*Shigella* | 31 | ns | ns | 0.39 | ns | 0.06 | 0.01 | 0.03 | 0.08 | 0 | 0.22 |
| OTU166 | *Turicibacter* | 31 | ns | -0.40 | ns | ns | 0.08 | 0.02 | 0.04 | 0.11 | 0 | 0.23 |
| OTU174 | *Turicibacter* | 31 | ns | -0.51 | ns | -0.42 | 0.07 | 0.02 | 0.04 | 0.10 | 0 | 0.24 |
| OTU209 | *Turicibacter* | 31 | ns | -0.49 | ns | -0.43 | 0.06 | 0.01 | 0.03 | 0.08 | 0 | 0.20 |
| OTU212 | *Enterobacter* | 31 | -0.40 | ns | ns | ns | 0.02 | 0.01 | 0.002 | 0.04 | 0 | 0.19 |
| OTU220 | *Escherichia*/*Hafnia*/*Shigella* | 31 | ns | ns | 0.36 | ns | 0.03 | 0.01 | 0.01 | 0.04 | 0 | 0.09 |
| OTU253 | *Turicibacter* | 31 | ns | -0.51 | ns | -0.36 | 0.04 | 0.01 | 0.02 | 0.06 | 0 | 0.14 |
| OTU276 | *Turicibacter* | 31 | ns | -0.49 | ns | ns | 0.03 | 0.01 | 0.02 | 0.05 | 0 | 0.10 |
| OTU298 | *Escherichia*/*Hafnia*/*Shigella* | 31 | ns | 0.30 | 0.38 | ns | 0.02 | 0.003 | 0.01 | 0.02 | 0 | 0.06 |
| OTU323 | *Turicibacter* | 31 | ns | -0.52 | ns | ns | 0.02 | 0.01 | 0.01 | 0.04 | 0 | 0.08 |
| OTU352 | *Turicibacter* | 31 | ns | -0.42 | ns | ns | 0.03 | 0.01 | 0.01 | 0.04 | 0 | 0.09 |
|  |  |  |  |  |  |  |  |  |  |  |  |  |
| Ceca |  |  |  |  |  |  |  |  |  |  |  |  |
| OTU4 | *Lactobacillus* | 31 | ns | ns | -0.40 | ns | 0.08 | 0.03 | 0.02 | 0.15 | 0 | 0.60 |
| OTU5 | [*Clostridium*] | 31 | ns | ns | ns | -0.36 | 5.27 | 1.09 | 3.04 | 7.49 | 0.11 | 17.18 |
| OTU6 | [*Acetanaerobacterium*] | 31 | ns | ns | ns | -0.53 | 6.58 | 1.24 | 4.05 | 9.12 | 0.004 | 20.85 |
| OTU7 | *Anaerotruncus* | 31 | ns | ns | 0.43 | -0.36 | 4.48 | 1.07 | 2.30 | 6.66 | 0.03 | 19.70 |
| OTU8 | *Lactobacillus* | 31 | ns | ns | -0.40 | ns | 0.05 | 0.02 | 0.01 | 0.08 | 0 | 0.38 |
| OTU17 | [*Clostridium*] | 31 | 0.41 | ns | ns | 0.49 | 3.10 | 0.66 | 1.74 | 4.45 | 0 | 9.06 |
| OTU19 | [*Clostridium*] | 31 | ns | -0.43 | ns | ns | 1.77 | 0.39 | 0.97 | 2.57 | 0.002 | 6.00 |
| OTU22 | [*Clostridium*] | 31 | ns | ns | ns | -0.40 | 1.71 | 0.59 | 0.51 | 2.91 | 0 | 4.76 |
| OTU23 | [*Clostridium*] | 31 | ns | 0.38 | ns | 0.61* | 1.01 | 0.39 | 0.22 | 1.80 | 0.002 | 5.02 |
| OTU32 | [*Clostridium*] | 31 | 0.38 | 0.39 | ns | 0.68* | 0.65 | 0.24 | 0.15 | 1.14 | 0 | 3.31 |
| OTU33 | [*Ethanologenbacterium*] | 31 | ns | ns | ns | -0.47 | 1.24 | 0.22 | 0.79 | 1.69 | 0.02 | 4.08 |
| OTU46 | *Faecalibacterium* | 31 | ns | ns | ns | 0.51 | 0.62 | 0.20 | 0.22 | 1.03 | 0 | 2.64 |
| OTU61 | Unclassified *Clostridiaceae* | 31 | ns | ns | ns | -0.39 | 0.45 | 0.17 | 0.09 | 0.80 | 0 | 2.40 |
| OTU69 | [*Clostridium*] | 31 | ns | -0.48 | ns | ns | 0.24 | 0.06 | 0.12 | 0.36 | 0 | 1.16 |
| OTU73 | [*Acetanaerobacterium*] | 31 | ns | ns | ns | 0.38 | 0.28 | 0.06 | 0.17 | 0.39 | 0.004 | 0.96 |
| OTU75 | [*Clostridium*] | 31 | ns | ns | ns | 0.59* | 0.29 | 0.05 | 0.18 | 0.39 | 0 | 1.02 |
| OTU94 | *Clostridium* | 31 | ns | ns | 0.47 | -0.40 | 0.21 | 0.05 | 0.11 | 0.31 | 0.004 | 0.80 |
| OTU108 | [*Clostridium*] | 31 | ns | -0.38 | ns | -0.37 | 0.17 | 0.04 | 0.08 | 0.25 | 0.004 | 0.54 |
| OTU117 | *Clostridium* | 31 | ns | ns | 0.50 | ns | 0.19 | 0.06 | 0.06 | 0.32 | 0 | 0.87 |
| OTU131 | [*Oscillibacter*] | 31 | ns | ns | ns | 0.36 | 0.13 | 0.02 | 0.09 | 0.18 | 0.004 | 0.37 |
| OTU141 | *Clostridium* | 31 | ns | ns | -0.36 | 0.47 | 0.14 | 0.03 | 0.07 | 0.21 | 0.01 | 0.61 |
| OTU142 | [*Clostridium*] | 31 | ns | ns | ns | -0.39 | 0.11 | 0.04 | 0.03 | 0.19 | 0 | 0.30 |
| OTU150 | *Clostridium* | 31 | ns | ns | ns | 0.37 | 0.13 | 0.02 | 0.08 | 0.18 | 0 | 0.42 |
| OTU161 | Unclassified *Clostridiaceae* | 31 | ns | ns | -0.53 | 0.49 | 0.05 | 0.02 | 0.005 | 0.10 | 0 | 0.25 |
| OTU164 | *Clostridium* | 31 | ns | ns | 0.39 | ns | 0.09 | 0.02 | 0.06 | 0.13 | 0 | 0.22 |
| OTU165 | [*Spiroplasma*] | 31 | ns | ns | -0.41 | 0.47 | 0.09 | 0.03 | 0.03 | 0.14 | 0 | 0.49 |
| OTU167 | [*Dehalobacterium*] | 31 | ns | ns | 0.44 | ns | 0.09 | 0.01 | 0.07 | 0.12 | 0.004 | 0.23 |
| OTU173 | [*Clostridium*] | 31 | ns | 0.36 | ns | 0.48 | 0.09 | 0.02 | 0.06 | 0.12 | 0.01 | 0.33 |
| OTU179 | [*Clostridium*] | 31 | ns | -0.36 | ns | ns | 0.07 | 0.02 | 0.04 | 0.10 | 0 | 0.26 |
| OTU182 | *Clostridium* | 31 | ns | ns | ns | 0.36 | 0.08 | 0.03 | 0.02 | 0.14 | 0 | 0.60 |
| OTU188 | [*Clostridium*] | 31 | 0.40 | ns | ns | 0.60* | 0.06 | 0.02 | 0.02 | 0.09 | 0 | 0.30 |
| OTU190 | *Eubacterium* | 31 | ns | ns | ns | 0.42 | 0.07 | 0.02 | 0.03 | 0.10 | 0.002 | 0.29 |
| OTU192 | [*Clostridium*] | 31 | ns | ns | ns | 0.38 | 0.06 | 0.01 | 0.04 | 0.08 | 0 | 0.15 |
| OTU205 | *Hespellia* | 31 | ns | ns | ns | -0.39 | 0.04 | 0.01 | 0.02 | 0.05 | 0.002 | 0.13 |
| OTU210 | [*Acetanaerobacterium*] | 31 | ns | -0.39 | ns | -0.63* | 0.06 | 0.01 | 0.04 | 0.08 | 0 | 0.19 |
| OTU217 | [*Clostridium*] | 31 | ns | ns | ns | -0.37 | 0.04 | 0.01 | 0.02 | 0.07 | 0 | 0.23 |
| OTU224 | [*Clostridium*] | 31 | ns | ns | -0.54 | 0.48 | 0.06 | 0.02 | 0.01 | 0.10 | 0 | 0.48 |
| OTU228 | [*Spiroplasma*] | 31 | 0.37 | ns | ns | ns | 0.07 | 0.02 | 0.03 | 0.11 | 0 | 0.29 |
| OTU233 | [*Clostridium*] | 31 | -0.40 | ns | ns | ns | 0.05 | 0.01 | 0.02 | 0.07 | 0 | 0.20 |
| OTU295 | [*Clostridium*] | 31 | ns | -0.39 | ns | -0.37 | 0.03 | 0.01 | 0.02 | 0.04 | 0 | 0.07 |
| OTU308 | [*Clostridium*] | 31 | ns | ns | ns | -0.51 | 0.03 | 0.01 | 0.02 | 0.04 | 0 | 0.11 |
| OTU325 | [*Clostridium*] | 31 | ns | ns | ns | -0.40 | 0.03 | 0.004 | 0.02 | 0.03 | 0 | 0.06 |
| OTU329 | *Clostridium* | 31 | ns | 0.38 | ns | ns | 0.04 | 0.01 | 0.02 | 0.06 | 0 | 0.12 |
| OTU341 | *Clostridium* | 31 | ns | ns | ns | 0.45 | 0.03 | 0.007 | 0.02 | 0.04 | 0.002 | 0.13 |
| OTU343 | *Anaerotruncus* | 31 | ns | ns | 0.46 | -0.39 | 0.03 | 0.01 | 0.02 | 0.04 | 0 | 0.09 |
| OTU348 | *Ruminococcus* | 31 | ns | ns | 0.43 | ns | 0.03 | 0.01 | 0.02 | 0.04 | 0 | 0.07 |
| OTU349 | *Sporobacter* | 31 | ns | 0.37 | ns | 0.53 | 0.02 | 0.003 | 0.02 | 0.03 | 0.004 | 0.05 |
| OTU362 | [*Clostridium*] | 31 | ns | -0.54 | ns | -0.63* | 0.02 | 0.005 | 0.01 | 0.03 | 0 | 0.08 |
| OTU415 | [*Clostridium*] | 31 | ns | 0.38 | ns | ns | 0.02 | 0.005 | 0.01 | 0.03 | 0 | 0.10 |
| OTU437 | [*Clostridium*] | 31 | ns | ns | 0.39 | -0.38 | 0.02 | 0.004 | 0.01 | 0.03 | 0 | 0.09 |
| OTU456 | [*Photorhabdus*] | 31 | ns | ns | -0.38 | 0.38 | 0.01 | 0.003 | 0.004 | 0.02 | 0 | 0.05 |
| OTU459 | [*Clostridium*] | 31 | ns | -0.51 | ns | ns | 0.01 | 0.003 | 0.01 | 0.02 | 0 | 0.05 |
| OTU466 | [*Clostridium*] | 31 | ns | ns | 0.37 | ns | 0.02 | 0.01 | 0.004 | 0.03 | 0 | 0.10 |
| OTU469 | [*Clostridium*] | 31 | ns | ns | ns | -0.40 | 0.01 | 0.003 | 0.01 | 0.02 | 0 | 0.05 |
| OTU500 | [*Clostridium*] | 31 | ns | ns | ns | -0.37 | 0.02 | 0.004 | 0.01 | 0.02 | 0 | 0.04 |
| OTU504 | *Clostridium* | 31 | ns | 0.42 | ns | ns | 0.02 | 0.004 | 0.01 | 0.03 | 0 | 0.08 |
| OTU519 | *Ruminococcus* | 31 | ns | 0.46 | ns | ns | 0.01 | 0.003 | 0.005 | 0.02 | 0 | 0.04 |
| OTU520 | [*Clostridium*] | 31 | ns | -0.65* | ns | -0.46 | 0.01 | 0.003 | 0.01 | 0.02 | 0 | 0.05 |
| OTU553 | [*Clostridium*] | 31 | 0.48 | ns | -0.39 | 0.66* | 0.02 | 0.004 | 0.01 | 0.02 | 0 | 0.09 |
| OTU610 | [*Clostridium*] | 31 | ns | ns | ns | 0.44 | 0.02 | 0.005 | 0.01 | 0.03 | 0 | 0.08 |
| OTU634 | [*Clostridium*] | 31 | ns | ns | ns | -0.37 | 0.01 | 0.002 | 0.01 | 0.02 | 0 | 0.04 |
| OTU725 | *Clostridium* | 31 | ns | 0.38 | ns | ns | 0.01 | 0.002 | 0.01 | 0.02 | 0 | 0.04 |
|  |  |  |  |  |  |  |  |  |  |  |  |  |
| Feces |  |  |  |  |  |  |  |  |  |  |  |  |
| OTU4 | *Lactobacillus* | 32 | 0.42 | ns | -0.37 | 0.50 | 1.17 | 0.65 | -0.16 | 2.49 | 0 | 7.80 |
| OTU5 | [*Clostridium*] | 32 | ns | ns | ns | -0.44 | 1.41 | 0.53 | 0.34 | 2.48 | 0.006 | 7.40 |
| OTU8 | *Lactobacillus* | 32 | 0.40 | ns | -0.39 | 0.51 | 0.59 | 0.32 | -0.06 | 1.24 | 0 | 3.58 |
| OTU18 | [*Blautia*] | 32 | ns | ns | ns | -0.36 | 0.35 | 0.10 | 0.13 | 0.56 | 0 | 2.02 |
| OTU19 | [*Clostridium*] | 32 | ns | ns | ns | -0.39 | 0.38 | 0.15 | 0.08 | 0.68 | 0 | 2.42 |
| OTU22 | [*Clostridium*] | 32 | ns | ns | 0.43 | -0.47 | 0.56 | 0.16 | 0.23 | 0.88 | 0 | 2.52 |
| OTU25 | [*Clostridium*] | 32 | ns | ns | 0.45 | ns | 0.28 | 0.09 | 0.09 | 0.47 | 0 | 1.91 |
| OTU38 | [*Clostridium*] | 32 | -0.37 | ns | ns | ns | 0.13 | 0.06 | 0.01 | 0.24 | 0 | 0.92 |
| OTU40 | *Turicibacter* | 32 | ns | -0.36 | ns | -0.38 | 0.10 | 0.05 | -0.002 | 0.20 | 0 | 0.93 |
| OTU52 | [*Clostridium*] | 32 | ns | ns | ns | -0.43 | 0.05 | 0.01 | 0.02 | 0.08 | 0 | 0.24 |
| OTU99 | [*Clostridium*] | 32 | ns | -0.37 | ns | ns | 0.04 | 0.02 | -0.003 | 0.08 | 0 | 0.27 |
| OTU173 | [*Clostridium*] | 32 | ns | ns | ns | -0.40 | 0.02 | 0.01 | 0.01 | 0.03 | 0 | 0.10 |
| OTU259 | [*Escherichia/Hafnia/Shigella*] | 32 | ns | -0.37 | ns | ns | 0.03 | 0.01 | 0.01 | 0.05 | 0 | 0.13 |
| OTU294 | [*Escherichia/Hafnia/Shigella*] | 32 | ns | ns | ns | -0.36 | 0.02 | 0.004 | 0.01 | 0.03 | 0 | 0.07 |

^a^Statistical comparisons were made for those OTUs that showed a relative abundance > 0.01% per intestinal site.

^b^Only significant (*P* ≤ 0.05) correlations are presented. **P* ≤ 0.001.

^c^ns, not significant; RFI, residual feed intake; TFI, total feed intake; TBWG, total body weight gain; FCR, feed conversion ratio; SE, standard error; CI, confidence interval; Pctl, percentile.

^d^Taxonomic classification based on the Greengenes 16S rRNA gene database (greengenes.lbl.gov/cgi-bin/nph-index.cgi).

^e^Sequences not distinguishable between *Escherichia*, *Hafnia* and *Shigella*.
