# Supplementary material for: Intestinal microbiota profiles associated with low and high residual feed intake in chickens across two geographical locations
Source: PLoS One. 2017 Nov 15;12(11):e0187766. doi: 10.1371/journal.pone.0187766 (PMC5687768; doi:10.1371/journal.pone.0187766)
Supplement: S4 Table — (DOCX) [file pone.0187766.s004.docx]

S4 Table. Most abundant operational taxonomic units (OTU) correlating to feed efficiency and performance traits in male chickens across two geographical locations and by intestinal site.

| OTU^a-c^ | Taxonomy (Genus)^d,e^ | n | RFI | TFI | TBWG | FCR | Mean | SE | Lower 95% CI | Upper 95% CI | 5th Pctl | 95th Pctl |
| --- | --- | --- | --- | --- | --- | --- | --- | --- | --- | --- | --- | --- |
| Ileum |  |  |  |  |  |  |  |  |  |  |  |  |
| OTU18 | [*Blautia*] | 37 | ns | ns | ns | 0.35 | 0.09 | 0.04 | 0.01 | 0.17 | 0 | 0.92 |
| OTU54 | *Enterobacter* | 37 | ns | ns | -0.40 | ns | 1.08 | 0.72 | -0.38 | 2.54 | 0 | 13.13 |
| OTU125 | *Enterococcus* | 37 | ns | ns | -0.36 | ns | 0.56 | 0.37 | -0.19 | 1.30 | 0 | 8.56 |
| OTU619 | *Pseudidiomarina* | 37 | ns | ns | -0.40 | ns | 0.01 | 0.01 | 0.002 | 0.02 | 0 | 0.04 |
|  |  |  |  |  |  |  |  |  |  |  |  |  |
| Ceca |  |  |  |  |  |  |  |  |  |  |  |  |
| OTU7 | *Anaerotruncus* | 37 | ns | ns | 0.37 | ns | 4.80 | 1.15 | 2.46 | 7.13 | 0.06 | 19.46 |
| OTU14 | [*Clostridium*] | 37 | ns | -0.34 | ns | ns | 1.94 | 0.45 | 1.02 | 2.86 | 0.002 | 6.16 |
| OTU29 | [*Clostridium*] | 37 | ns | ns | ns | 0.47 | 1.93 | 0.75 | 0.41 | 3.45 | 0 | 12.93 |
| OTU32 | [*Clostridium*] | 37 | ns | ns | -0.43 | ns | 1.05 | 0.27 | 0.50 | 1.59 | 0 | 5.33 |
| OTU43 | *Eubacterium* | 37 | ns | -0.33 | ns | ns | 0.80 | 0.23 | 0.33 | 1.28 | 0.02 | 3.05 |
| OTU49 | [*Clostridium*] | 37 | ns | ns | ns | 0.35 | 0.95 | 0.23 | 0.47 | 1.42 | 0 | 4.53 |
| OTU61 | Unclassified *Clostridiaceae* | 37 | ns | ns | ns | -0.37 | 0.39 | 0.14 | 0.11 | 0.67 | 0 | 3.42 |
| OTU62 | *Streptococcus* | 37 | ns | ns | ns | -0.38 | 0.04 | 0.01 | 0.02 | 0.06 | 0 | 0.18 |
| OTU84 | *Eubacterium* | 37 | 0.37 | 0.46 | ns | ns | 0.25 | 0.07 | 0.11 | 0.39 | 0.01 | 0.96 |
| OTU87 | [*Clostridium*] | 37 | ns | ns | ns | -0.42 | 0.29 | 0.07 | 0.16 | 0.43 | 0 | 1.28 |
| OTU91 | [*Heliorestis*] | 37 | ns | ns | ns | 0.36 | 0.32 | 0.12 | 0.08 | 0.56 | 0 | 1.44 |
| OTU94 | *Clostridium* | 37 | ns | ns | ns | -0.35 | 0.23 | 0.05 | 0.13 | 0.33 | 0 | 1.01 |
| OTU99 | [*Clostridium*] | 37 | ns | ns | 0.45 | ns | 0.23 | 0.06 | 0.12 | 0.34 | 0 | 0.94 |
| OTU108 | [*Clostridium*] | 37 | ns | ns | 0.37 | ns | 0.19 | 0.03 | 0.14 | 0.25 | 0.01 | 0.51 |
| OTU128 | [*Clostridium*] | 37 | ns | ns | 0.34 | ns | 0.15 | 0.04 | 0.07 | 0.22 | 0 | 0.78 |
| OTU141 | *Clostridium* | 37 | ns | ns | -0.38 | ns | 0.11 | 0.02 | 0.07 | 0.16 | 0.01 | 0.38 |
| OTU152 | [*Clostridium*] | 37 | ns | ns | ns | 0.44 | 0.15 | 0.04 | 0.07 | 0.22 | 0 | 0.93 |
| OTU157 | [*Oscillibacter*] | 37 | ns | ns | ns | -0.34 | 0.10 | 0.03 | 0.04 | 0.15 | 0 | 0.32 |
| OTU161 | Unclassified *Clostridiaceae* | 37 | ns | ns | -0.37 | ns | 0.16 | 0.05 | 0.07 | 0.25 | 0 | 0.78 |
| OTU167 | [*Dehalobacterium*] | 37 | ns | ns | 0.34 | ns | 0.08 | 0.01 | 0.06 | 0.11 | 0 | 0.23 |
| OTU188 | [*Clostridium*] | 37 | ns | ns | -0.35 | ns | 0.08 | 0.02 | 0.05 | 0.11 | 0 | 0.32 |
| OTU205 | *Hespellia* | 37 | ns | ns | 0.33 | -0.46 | 0.03 | 0.01 | 0.02 | 0.04 | 0 | 0.10 |
| OTU210 | [*Acetanaerobacterium*] | 37 | ns | ns | ns | -0.40 | 0.06 | 0.01 | 0.03 | 0.08 | 0 | 0.24 |
| OTU237 | *Anaerotruncus* | 37 | ns | ns | 0.39 | -0.34 | 0.06 | 0.01 | 0.03 | 0.09 | 0 | 0.27 |
| OTU238 | *Eubacterium* | 37 | ns | -0.37 | ns | ns | 0.04 | 0.01 | 0.02 | 0.07 | 0 | 0.24 |
| OTU252 | [*Eubacterium*] | 37 | ns | -0.33 | -0.45 | ns | 0.04 | 0.01 | 0.02 | 0.06 | 0 | 0.11 |
| OTU274 | [*Ruminococcus*] | 37 | ns | ns | 0.44 | -0.40 | 0.04 | 0.01 | 0.02 | 0.07 | 0 | 0.21 |
| OTU281 | *Clostridium* | 37 | 0.41 | ns | ns | ns | 0.01 | 0.002 | 0.01 | 0.02 | 0 | 0.05 |
| OTU285 | [*Clostridium*] | 37 | ns | ns | 0.33 | -0.42 | 0.04 | 0.01 | 0.02 | 0.06 | 0 | 0.17 |
| OTU289 | [*Clostridium*] | 37 | 0.38 | ns | 0.43 | ns | 0.03 | 0.01 | 0.02 | 0.04 | 0 | 0.10 |
| OTU292 | Unclassified *Clostridiaceae* | 37 | ns | ns | 0.33 | ns | 0.04 | 0.01 | 0.02 | 0.06 | 0 | 0.18 |
| OTU308 | [*Clostridium*] | 37 | ns | ns | ns | -0.34 | 0.03 | 0.01 | 0.02 | 0.05 | 0 | 0.12 |
| OTU343 | *Anaerotruncus* | 37 | ns | ns | 0.34 | -0.36 | 0.03 | 0.01 | 0.01 | 0.04 | 0 | 0.15 |
| OTU346 | [*Anaerotruncus*] | 37 | ns | ns | 0.38 | ns | 0.03 | 0.01 | 0.02 | 0.05 | 0 | 0.15 |
| OTU362 | [*Clostridium*] | 37 | ns | ns | ns | -0.37 | 0.02 | 0.005 | 0.01 | 0.03 | 0 | 0.07 |
| OTU368 | *Ruminococcus* | 37 | ns | ns | ns | -0.34 | 0.02 | 0.004 | 0.01 | 0.02 | 0 | 0.07 |
| OTU388 | [*Clostridium*] | 37 | ns | ns | 0.37 | ns | 0.02 | 0.004 | 0.01 | 0.03 | 0 | 0.10 |
| OTU392 | [*Clostridium*] | 37 | ns | -0.33 | ns | ns | 0.02 | 0.003 | 0.01 | 0.03 | 0 | 0.06 |
| OTU470 | [*Clostridium*] | 37 | ns | ns | -0.38 | ns | 0.02 | 0.002 | 0.01 | 0.02 | 0 | 0.04 |
| OTU482 | [*Clostridium*] | 37 | ns | ns | -0.45 | ns | 0.02 | 0.004 | 0.01 | 0.02 | 0 | 0.08 |
| OTU507 | [*Clostridium*] | 37 | ns | ns | 0.38 | ns | 0.01 | 0.003 | 0.01 | 0.02 | 0 | 0.06 |
| OTU515 | [*Clostridium*] | 37 | 0.33 | ns | ns | ns | 0.02 | 0.003 | 0.01 | 0.02 | 0 | 0.05 |
| OTU574 | *Eubacterium* | 37 | 0.59* | 0.41 | ns | ns | 0.01 | 0.002 | 0.01 | 0.02 | 0 | 0.03 |
| OTU654 | [*Ruminococcus*] | 37 | ns | -0.34 | ns | ns | 0.01 | 0.002 | 0.01 | 0.01 | 0 | 0.04 |
|  |  |  |  |  |  |  |  |  |  |  |  |  |
| Feces |  |  |  |  |  |  |  |  |  |  |  |  |
| OTU5 | [*Clostridium*] | 37 | ns | ns | ns | -0.40 | 2.22 | 0.77 | 0.66 | 3.79 | 0.003 | 15.19 |
| OTU12 | [*Clostridium*] | 37 | -0.34 | ns | ns | ns | 0.96 | 0.25 | 0.45 | 1.47 | 0.004 | 4.67 |
| OTU22 | [*Clostridium*] | 37 | ns | ns | ns | -0.37 | 0.41 | 0.16 | 0.08 | 0.74 | 0 | 3.77 |
| OTU31 | [*Spiroplasma*] | 37 | -0.35 | ns | ns | ns | 0.23 | 0.06 | 0.11 | 0.36 | 0 | 0.91 |
| OTU32 | [*Clostridium*] | 37 | ns | ns | -0.33 | ns | 0.74 | 0.37 | -0.01 | 1.49 | 0 | 8.35 |
| OTU33 | [*Ethanologenbacterium*] | 37 | ns | ns | ns | -0.34 | 0.10 | 0.03 | 0.03 | 0.17 | 0 | 0.65 |
| OTU40 | *Turicibacter* | 37 | ns | ns | ns | -0.37 | 0.11 | 0.06 | -0.01 | 0.23 | 0 | 1.52 |
| OTU55 | [*Clostridium*] | 37 | -0.36 | ns | ns | ns | 0.16 | 0.05 | 0.05 | 0.27 | 0 | 0.97 |
| OTU77 | *Clostridium* | 37 | ns | ns | ns | -0.39 | 0.04 | 0.01 | 0.02 | 0.06 | 0 | 0.25 |
| OTU94 | *Clostridium* | 37 | ns | ns | ns | -0.43 | 0.05 | 0.02 | 0.02 | 0.09 | 0 | 0.38 |
| OTU103 | *Clostridium* | 37 | ns | ns | ns | -0.37 | 0.06 | 0.02 | 0.03 | 0.10 | 0 | 0.38 |
| OTU104 | [*Clostridium*] | 37 | -0.37 | ns | ns | ns | 0.07 | 0.02 | 0.03 | 0.11 | 0 | 0.39 |
| OTU125 | *Enterococcus* | 37 | ns | ns | -0.37 | ns | 0.15 | 0.10 | -0.06 | 0.35 | 0 | 0.88 |
| OTU126 | *Eubacterium* | 37 | ns | ns | ns | -0.33 | 0.05 | 0.02 | 0.01 | 0.10 | 0 | 0.38 |
| OTU143 | [*Clostridium*] | 37 | -0.34 | ns | ns | ns | 0.02 | 0.01 | 0.002 | 0.03 | 0 | 0.17 |
| OTU145 | *Oscillibacter* | 37 | ns | ns | ns | -0.37 | 0.03 | 0.01 | 0.02 | 0.05 | 0 | 0.15 |
| OTU151 | [*Clostridium*] | 37 | ns | ns | ns | -0.35 | 0.04 | 0.01 | 0.02 | 0.06 | 0 | 0.21 |
| OTU167 | [*Dehalobacterium*] | 37 | ns | ns | ns | -0.35 | 0.01 | 0.003 | 0.005 | 0.02 | 0 | 0.05 |
| OTU203 | *Anaerostipes* | 37 | ns | ns | ns | 0.38 | 0.04 | 0.03 | -0.02 | 0.10 | 0 | 0.25 |
| OTU205 | *Hespellia* | 37 | ns | ns | ns | -0.33 | 0.03 | 0.01 | 0.01 | 0.05 | 0 | 0.26 |
| OTU207 | *Hespellia* | 37 | ns | ns | ns | -0.34 | 0.03 | 0.01 | 0.01 | 0.04 | 0 | 0.17 |
| OTU215 | *Clostridium* | 37 | -0.42 | ns | ns | ns | 0.01 | 0.004 | 0.01 | 0.02 | 0 | 0.05 |
| OTU224 | [*Clostridium*] | 37 | -0.34 | ns | ns | ns | 0.02 | 0.004 | 0.01 | 0.02 | 0 | 0.06 |
| OTU259 | [*Escherichia/Hafnia/Shigella*] | 37 | -0.33 | ns | ns | ns | 0.02 | 0.005 | 0.01 | 0.03 | 0 | 0.08 |
| OTU288 | *Turicibacter* | 37 | -0.34 | ns | ns | ns | 0.01 | 0.004 | 0.01 | 0.02 | 0 | 0.08 |

^a^Statistical comparisons were made for those OTUs that showed a relative abundance > 0.01% per intestinal site.

^b^Only significant (*P* ≤ 0.05) correlations are presented. **P* ≤ 0.001.

^c^ns, not significant; RFI, residual feed intake; TFI, total feed intake; TBWG, total body weight gain; FCR, feed conversion ratio; SE, standard error; CI, confidence interval; Pctl, percentile.

^d^Taxonomic classification based on the Greengenes 16S rRNA gene database (greengenes.lbl.gov/cgi-bin/nph-index.cgi).

^e^Sequences not distinguishable between *Escherichia*, *Hafnia* and *Shigella*.
