# Supplementary material for: Intestinal microbiota profiles associated with low and high residual feed intake in chickens across two geographical locations
Source: PLoS One. 2017 Nov 15;12(11):e0187766. doi: 10.1371/journal.pone.0187766 (PMC5687768; doi:10.1371/journal.pone.0187766)
Supplement: S5 Table — (DOCX) [file pone.0187766.s005.docx]

S5 Table. Selected KEGG pathways correlating to feed efficiency and performance traits in female chickens across two geographical locations and by intestinal site.

| COG Pathway^a-c^ | KEGG Pathway | n | RFI | TFI | TBWG | FCR | Mean | SE | Lower 95% CI | Upper 95% CI | 5th Pctl | 95th Pctl |
| --- | --- | --- | --- | --- | --- | --- | --- | --- | --- | --- | --- | --- |
| Ileum |  |  |  |  |  |  |  |  |  |  |  |  |
| Amino acid metabolism | Amino acid metabolism | 31 | ns | ns | ns | -0.48 | 0.26 | 0.01 | 0.23 | 0.28 | 0.16 | 0.39 |
| Amino acid metabolism | Cysteine and methionine metabolism | 31 | ns | ns | ns | -0.45 | 0.96 | 0.02 | 0.91 | 1.00 | 0.70 | 1.10 |
| Amino acid metabolism | Glycine, serine and threonine metabolism | 31 | ns | ns | 0.36 | ns | 0.85 | 0.03 | 0.79 | 0.90 | 0.55 | 0.95 |
| Amino acid metabolism | Histidine metabolism | 31 | ns | ns | ns | -0.44 | 0.47 | 0.03 | 0.40 | 0.54 | 0.21 | 0.87 |
| Amino acid metabolism | Tyrosine metabolism | 31 | ns | -0.56 | ns | -0.49 | 0.43 | 0.01 | 0.41 | 0.45 | 0.36 | 0.56 |
| Amino acid metabolism | Valine, leucine and isoleucine biosynthesis | 31 | ns | 0.39 | 0.46 | ns | 0.60 | 0.03 | 0.54 | 0.66 | 0.30 | 0.83 |
| Biosynthesis of other secondary metabolites | Novobiocin biosynthesis | 31 | ns | ns | 0.41 | ns | 0.12 | 0.01 | 0.11 | 0.14 | 0.02 | 0.17 |
| Biosynthesis of other secondary metabolites | Phenylpropanoid biosynthesis | 31 | ns | -0.51 | ns | -0.46 | 0.08 | 0.01 | 0.06 | 0.10 | 0.01 | 0.22 |
| Carbohydrate metabolism | C5-Branched dibasic acid metabolism | 31 | ns | ns | 0.41 | ns | 0.26 | 0.02 | 0.23 | 0.30 | 0.05 | 0.35 |
| Carbohydrate metabolism | Carbohydrate metabolism | 31 | ns | -0.56 | ns | ns | 0.13 | 0.01 | 0.12 | 0.15 | 0.06 | 0.21 |
| Carbohydrate metabolism | Glycolysis / Gluconeogenesis | 31 | ns | ns | -0.39 | ns | 1.32 | 0.05 | 1.22 | 1.42 | 1.02 | 1.82 |
| Carbohydrate metabolism | Pentose and glucuronate interconversions | 31 | ns | ns | ns | -0.37 | 0.44 | 0.03 | 0.38 | 0.49 | 0.24 | 0.72 |
| Carbohydrate metabolism | Pentose phosphate pathway | 31 | ns | -0.40 | ns | ns | 0.91 | 0.02 | 0.87 | 0.95 | 0.79 | 1.06 |
| Carbohydrate metabolism | Pyruvate metabolism | 31 | ns | ns | ns | -0.57* | 1.21 | 0.01 | 1.18 | 1.23 | 1.09 | 1.29 |
| Carbohydrate metabolism | Starch and sucrose metabolism | 31 | ns | -0.48 | -0.36 | ns | 0.88 | 0.04 | 0.80 | 0.96 | 0.48 | 1.22 |
| Cell motility | Bacterial chemotaxis | 31 | ns | 0.43 | ns | ns | 0.42 | 0.04 | 0.35 | 0.50 | 0.07 | 0.73 |
| Cell motility | Flagellar assembly | 31 | ns | 0.47 | 0.40 | ns | 0.54 | 0.05 | 0.43 | 0.65 | 0.06 | 1.10 |
| Digestive system | Peptidases | 31 | ns | ns | -0.39 | ns | 1.95 | 0.05 | 1.84 | 2.05 | 1.66 | 2.52 |
| Energy metabolism | Oxidative phosphorylation | 31 | ns | 0.49 | ns | 0.39 | 1.16 | 0.05 | 1.06 | 1.27 | 0.84 | 1.65 |
| Energy metabolism | Sulfur metabolism | 31 | ns | ns | 0.40 | ns | 0.29 | 0.01 | 0.27 | 0.32 | 0.20 | 0.38 |
| Folding, sorting and degradation | Chaperones and folding catalysts | 31 | ns | 0.47 | ns | 0.44 | 1.11 | 0.02 | 1.08 | 1.15 | 0.95 | 1.26 |
| Glycan biosynthesis and metabolism | Glycosaminoglycan degradation | 31 | ns | ns | -0.38 | ns | 0.01 | 0.003 | 0.01 | 0.02 | 0.002 | 0.05 |
| Glycan biosynthesis and metabolism | Other glycan degradation | 31 | ns | -0.50 | ns | -0.38 | 0.06 | 0.01 | 0.05 | 0.07 | 0.02 | 0.14 |
| Infectious diseases: Bacterial | Vibrio cholerae pathogenic cycle | 31 | ns | ns | 0.38 | ns | 0.11 | 0.01 | 0.10 | 0.13 | 0.05 | 0.18 |
| Lipid metabolism | Arachidonic acid metabolism | 31 | ns | -0.45 | ns | -0.45 | 0.05 | 0.01 | 0.04 | 0.06 | 0.01 | 0.13 |
| Lipid metabolism | Fatty acid biosynthesis | 31 | ns | ns | 0.47 | ns | 0.48 | 0.01 | 0.45 | 0.51 | 0.34 | 0.61 |
| Lipid metabolism | Glycerolipid metabolism | 31 | ns | -0.46 | -0.36 | ns | 0.45 | 0.01 | 0.42 | 0.47 | 0.35 | 0.54 |
| Lipid metabolism | Linoleic acid metabolism | 31 | ns | -0.37 | ns | ns | 0.04 | 0.01 | 0.02 | 0.05 | 0.003 | 0.13 |
| Lipid metabolism | Lipid biosynthesis proteins | 31 | ns | ns | 0.39 | ns | 0.60 | 0.01 | 0.58 | 0.63 | 0.46 | 0.71 |
| Lipid metabolism | Lipid metabolism | 31 | ns | -0.42 | ns | ns | 0.16 | 0.01 | 0.15 | 0.17 | 0.09 | 0.21 |
| Lipid metabolism | Synthesis and degradation of ketone bodies | 31 | ns | ns | -0.41 | ns | 0.08 | 0.01 | 0.07 | 0.10 | 0.04 | 0.16 |
| Membrane transport | ABC transporters | 31 | ns | -0.41 | ns | -0.42 | 4.39 | 0.10 | 4.20 | 4.59 | 3.73 | 5.41 |
| Membrane transport | Cell motility and secretion | 31 | ns | ns | ns | 0.48 | 0.27 | 0.01 | 0.25 | 0.30 | 0.16 | 0.36 |
| Membrane transport | Secretion system | 31 | ns | 0.36 | ns | ns | 2.13 | 0.09 | 1.95 | 2.31 | 1.19 | 2.76 |
| Membrane transport | Transporters | 31 | ns | -0.52 | ns | ns | 8.28 | 0.17 | 7.93 | 8.63 | 6.51 | 9.54 |
| Metabolism of cofactors and vitamins | Biotin metabolism | 31 | ns | ns | 0.39 | ns | 0.13 | 0.01 | 0.11 | 0.15 | 0.02 | 0.22 |
| Metabolism of cofactors and vitamins | Folate biosynthesis | 31 | ns | ns | 0.40 | ns | 0.38 | 0.01 | 0.35 | 0.41 | 0.25 | 0.51 |
| Metabolism of cofactors and vitamins | Pantothenate and CoA biosynthesis | 31 | ns | 0.48 | 0.42 | ns | 0.54 | 0.02 | 0.50 | 0.58 | 0.29 | 0.70 |
| Metabolism of cofactors and vitamins | Porphyrin and chlorophyll metabolism | 31 | ns | ns | 0.36 | ns | 0.71 | 0.05 | 0.60 | 0.81 | 0.25 | 1.20 |
| Metabolism of cofactors and vitamins | Retinol metabolism | 31 | ns | -0.46 | ns | ns | 0.05 | 0.004 | 0.04 | 0.06 | 0.01 | 0.09 |
| Metabolism of cofactors and vitamins | Vitamin B6 metabolism | 31 | ns | ns | 0.41 | ns | 0.19 | 0.01 | 0.17 | 0.21 | 0.08 | 0.26 |
| Metabolism of other amino acids | Cyanoamino acid metabolism | 31 | ns | -0.55 | ns | -0.43 | 0.25 | 0.02 | 0.22 | 0.29 | 0.15 | 0.51 |
| Metabolism of other amino acids | Phosphonate and phosphinate metabolism | 31 | ns | ns | 0.38 | ns | 0.05 | 0.004 | 0.04 | 0.06 | 0.01 | 0.09 |
| Metabolism of other amino acids | Selenocompound metabolism | 31 | ns | -0.36 | ns | -0.37 | 0.42 | 0.005 | 0.41 | 0.43 | 0.37 | 0.46 |
| Metabolism of other amino acids | Taurine and hypotaurine metabolism | 31 | ns | -0.54 | ns | ns | 0.15 | 0.01 | 0.13 | 0.17 | 0.11 | 0.29 |
| Metabolism of terpenoids and polyketides | Polyketide sugar unit biosynthesis | 31 | ns | -0.41 | ns | ns | 0.13 | 0.01 | 0.11 | 0.15 | 0.07 | 0.26 |
| Replication and repair | Replication, recombination and repair proteins | 31 | ns | 0.39 | ns | ns | 0.98 | 0.02 | 0.93 | 1.03 | 0.73 | 1.14 |
| Signaling molecules and interaction | Cellular antigens | 31 | ns | -0.59* | ns | -0.36 | 0.05 | 0.01 | 0.04 | 0.06 | 0.01 | 0.14 |
| Translation | RNA transport | 31 | ns | -0.41 | ns | ns | 0.18 | 0.01 | 0.16 | 0.20 | 0.11 | 0.31 |
| Translation | Ribosome biogenesis in eukaryotes | 31 | ns | ns | ns | 0.48 | 0.06 | 0.001 | 0.06 | 0.06 | 0.05 | 0.07 |
| Transport and catabolism | Lysosome | 31 | ns | ns | -0.37 | ns | 0.02 | 0.003 | 0.01 | 0.02 | 0.002 | 0.05 |
| Xenobiotics biodegradation and metabolism | Chloroalkane and chloroalkene degradation | 31 | ns | -0.47 | ns | ns | 0.19 | 0.01 | 0.17 | 0.21 | 0.12 | 0.33 |
| Xenobiotics biodegradation and metabolism | Drug metabolism - other enzymes | 31 | ns | -0.37 | ns | ns | 0.34 | 0.01 | 0.31 | 0.36 | 0.26 | 0.47 |
| Xenobiotics biodegradation and metabolism | Ethylbenzene degradation | 31 | ns | -0.42 | ns | -0.43 | 0.06 | 0.01 | 0.05 | 0.08 | 0.03 | 0.18 |
| Xenobiotics biodegradation and metabolism | Naphthalene degradation | 31 | ns | -0.45 | ns | ns | 0.17 | 0.01 | 0.15 | 0.20 | 0.11 | 0.33 |
| Xenobiotics biodegradation and metabolism | Nitrotoluene degradation | 31 | ns | ns | 0.38 | ns | 0.09 | 0.01 | 0.07 | 0.10 | 0.01 | 0.14 |
| Xenobiotics biodegradation and metabolism | Toluene degradation | 31 | ns | 0.41 | ns | 0.36 | 0.18 | 0.01 | 0.15 | 0.21 | 0.03 | 0.27 |
|  |  |  |  |  |  |  |  |  |  |  |  |  |
| Ceca |  |  |  |  |  |  |  |  |  |  |  |  |
| Amino acid metabolism | Lysine biosynthesis | 31 | -0.46 | ns | ns | -0.46 | 0.97 | 0.01 | 0.95 | 0.99 | 0.88 | 1.05 |
| Amino acid metabolism | Phenylalanine, tyrosine and tryptophan biosynthesis | 31 | -0.37 | ns | ns | ns | 0.85 | 0.01 | 0.84 | 0.87 | 0.79 | 0.94 |
| Biosynthesis of other secondary metabolites | Butirosin and neomycin biosynthesis | 31 | ns | ns | 0.37 | ns | 0.08 | 0.002 | 0.07 | 0.08 | 0.06 | 0.10 |
| Carbohydrate metabolism | C5-Branched dibasic acid metabolism | 31 | -0.40 | ns | ns | ns | 0.36 | 0.002 | 0.35 | 0.36 | 0.34 | 0.37 |
| Cell motility | Bacterial chemotaxis | 31 | ns | ns | ns | -0.39 | 0.72 | 0.02 | 0.67 | 0.77 | 0.51 | 0.94 |
| Energy metabolism | Carbon fixation pathways in prokaryotes | 31 | ns | -0.38 | ns | ns | 1.14 | 0.01 | 1.12 | 1.15 | 1.05 | 1.19 |
| Energy metabolism | Methane metabolism | 31 | ns | ns | 0.42 | -0.53 | 1.51 | 0.02 | 1.48 | 1.54 | 1.35 | 1.68 |
| Folding, sorting and degradation | Chaperones and folding catalysts | 31 | ns | ns | -0.38 | 0.38 | 1.01 | 0.01 | 0.99 | 1.03 | 0.92 | 1.10 |
| Folding, sorting and degradation | Protein folding and associated processing | 31 | ns | ns | ns | 0.41 | 0.58 | 0.01 | 0.57 | 0.60 | 0.52 | 0.67 |
| Lipid metabolism | Glycerophospholipid metabolism | 31 | ns | ns | ns | 0.52 | 0.60 | 0.01 | 0.58 | 0.62 | 0.52 | 0.69 |
| Lipid metabolism | Linoleic acid metabolism | 31 | ns | ns | 0.44 | -0.39 | 0.08 | 0.004 | 0.07 | 0.09 | 0.05 | 0.14 |
| Lipid metabolism | Lipid biosynthesis proteins | 31 | -0.37 | ns | ns | ns | 0.67 | 0.004 | 0.66 | 0.68 | 0.64 | 0.72 |
| Membrane transport | ABC transporters | 31 | ns | ns | 0.40 | -0.45 | 4.39 | 0.06 | 4.26 | 4.52 | 3.96 | 5.17 |
| Metabolism of cofactors and vitamins | Folate biosynthesis | 31 | ns | ns | ns | 0.45 | 0.29 | 0.01 | 0.28 | 0.31 | 0.25 | 0.36 |
| Metabolism of cofactors and vitamins | Lipoic acid metabolism | 31 | 0.37 | ns | ns | 0.51 | 0.03 | 0.002 | 0.02 | 0.03 | 0.01 | 0.05 |
| Metabolism of cofactors and vitamins | Nicotinate and nicotinamide metabolism | 31 | ns | ns | ns | 0.36 | 0.46 | 0.003 | 0.45 | 0.47 | 0.44 | 0.49 |
| Metabolism of cofactors and vitamins | Porphyrin and chlorophyll metabolism | 31 | ns | ns | 0.37 | -0.40 | 1.07 | 0.02 | 1.04 | 1.11 | 0.92 | 1.26 |
| Metabolism of cofactors and vitamins | Riboflavin metabolism | 31 | ns | ns | -0.45 | ns | 0.24 | 0.01 | 0.23 | 0.25 | 0.18 | 0.28 |
| Metabolism of other amino acids | Glutathione metabolism | 31 | ns | ns | -0.37 | 0.40 | 0.18 | 0.01 | 0.16 | 0.19 | 0.12 | 0.28 |
| Metabolism of other amino acids | Taurine and hypotaurine metabolism | 31 | ns | ns | -0.47 | 0.40 | 0.11 | 0.001 | 0.11 | 0.12 | 0.10 | 0.13 |
| Metabolism of other amino acids | beta-Alanine metabolism | 31 | ns | ns | ns | 0.49 | 0.14 | 0.01 | 0.13 | 0.15 | 0.10 | 0.21 |
| Metabolism of terpenoids and polyketides | Biosynthesis of ansamycins | 31 | ns | ns | 0.41 | -0.43 | 0.16 | 0.01 | 0.15 | 0.17 | 0.11 | 0.23 |
| Metabolism of terpenoids and polyketides | Terpenoid backbone biosynthesis | 31 | ns | -0.36 | ns | ns | 0.66 | 0.01 | 0.65 | 0.68 | 0.57 | 0.71 |
| Nucleotide metabolism | Nucleotide metabolism | 31 | ns | ns | -0.37 | ns | 0.06 | 0.002 | 0.06 | 0.07 | 0.04 | 0.09 |
| Replication and repair | DNA repair and recombination proteins | 31 | ns | ns | -0.36 | ns | 3.27 | 0.03 | 3.22 | 3.32 | 3.04 | 3.53 |
| Translation | Ribosome Biogenesis | 31 | ns | ns | -0.39 | ns | 1.69 | 0.01 | 1.66 | 1.71 | 1.55 | 1.80 |
| Translation | Ribosome biogenesis in eukaryotes | 31 | ns | ns | -0.44 | 0.48 | 0.06 | 0.001 | 0.06 | 0.06 | 0.05 | 0.06 |
| Xenobiotics biodegradation and metabolism | Benzoate degradation | 31 | ns | -0.37 | ns | ns | 0.30 | 0.004 | 0.29 | 0.31 | 0.25 | 0.33 |
| Xenobiotics biodegradation and metabolism | Bisphenol degradation | 31 | ns | ns | 0.45 | -0.38 | 0.09 | 0.004 | 0.08 | 0.10 | 0.06 | 0.15 |
| Xenobiotics biodegradation and metabolism | Chloroalkane and chloroalkene degradation | 31 | ns | ns | 0.45 | -0.39 | 0.24 | 0.01 | 0.22 | 0.25 | 0.18 | 0.30 |
| Xenobiotics biodegradation and metabolism | Ethylbenzene degradation | 31 | ns | ns | ns | -0.43 | 0.07 | 0.003 | 0.06 | 0.07 | 0.04 | 0.10 |
| Xenobiotics biodegradation and metabolism | Naphthalene degradation | 31 | ns | ns | 0.36 | -0.38 | 0.17 | 0.003 | 0.17 | 0.18 | 0.15 | 0.20 |
| Xenobiotics biodegradation and metabolism | Nitrotoluene degradation | 31 | ns | ns | 0.40 | -0.48 | 0.15 | 0.01 | 0.13 | 0.16 | 0.10 | 0.24 |
|  |  |  |  |  |  |  |  |  |  |  |  |  |
| Feces |  |  |  |  |  |  |  |  |  |  |  |  |
| Amino acid metabolism | Arginine and proline metabolism | 32 | ns | ns | 0.37 | ns | 1.11 | 0.03 | 1.05 | 1.16 | 0.89 | 1.41 |
| Amino acid metabolism | Phenylalanine, tyrosine and tryptophan biosynthesis | 32 | ns | ns | ns | -0.44 | 0.72 | 0.01 | 0.70 | 0.75 | 0.57 | 0.84 |
| Amino acid metabolism | Valine, leucine and isoleucine biosynthesis | 32 | ns | ns | ns | -0.36 | 0.70 | 0.01 | 0.67 | 0.73 | 0.56 | 0.87 |
| Biosynthesis of other secondary metabolites | Novobiocin biosynthesis | 32 | -0.35 | ns | ns | ns | 0.15 | 0.003 | 0.14 | 0.15 | 0.12 | 0.17 |
| Biosynthesis of other secondary metabolites | Tropane, piperidine and pyridine alkaloid biosynthesis | 32 | -0.38 | ns | ns | ns | 0.13 | 0.002 | 0.13 | 0.14 | 0.10 | 0.15 |
| Carbohydrate metabolism | C5-Branched dibasic acid metabolism | 32 | -0.41 | ns | 0.45 | -0.59* | 0.33 | 0.005 | 0.32 | 0.34 | 0.24 | 0.36 |
| Carbohydrate metabolism | Pyruvate metabolism | 32 | ns | ns | 0.37 | ns | 1.23 | 0.01 | 1.21 | 1.25 | 1.15 | 1.33 |
| Energy metabolism | Energy metabolism | 32 | ns | ns | ns | -0.41 | 1.00 | 0.01 | 0.98 | 1.02 | 0.86 | 1.10 |
| Lipid metabolism | Lipid biosynthesis proteins | 32 | ns | ns | ns | -0.37 | 0.63 | 0.01 | 0.62 | 0.65 | 0.57 | 0.70 |
| Lipid metabolism | Synthesis and degradation of ketone bodies | 32 | ns | ns | ns | 0.41 | 0.05 | 0.003 | 0.05 | 0.06 | 0.03 | 0.09 |
| Metabolism of cofactors and vitamins | Vitamin B6 metabolism | 32 | ns | ns | ns | -0.37 | 0.23 | 0.004 | 0.23 | 0.24 | 0.20 | 0.26 |
| Metabolism of other amino acids | D-Alanine metabolism | 32 | ns | ns | ns | 0.53 | 0.12 | 0.003 | 0.11 | 0.12 | 0.08 | 0.16 |
| Metabolism of other amino acids | D-Glutamine and D-glutamate metabolism | 32 | ns | ns | ns | 0.36 | 0.14 | 0.003 | 0.14 | 0.15 | 0.12 | 0.17 |
| Metabolism of other amino acids | Taurine and hypotaurine metabolism | 32 | ns | ns | ns | 0.44 | 0.13 | 0.002 | 0.13 | 0.13 | 0.11 | 0.14 |
| Metabolism of terpenoids and polyketides | Prenyltransferases | 32 | ns | ns | ns | 0.37 | 0.28 | 0.01 | 0.26 | 0.29 | 0.20 | 0.36 |
| Signal transduction | Signal transduction mechanisms | 32 | ns | ns | -0.37 | 0.37 | 0.65 | 0.01 | 0.63 | 0.67 | 0.58 | 0.72 |
| Signaling molecules and interaction | Ion channels | 32 | 0.38 | ns | ns | 0.40 | 0.03 | 0.002 | 0.03 | 0.04 | 0.01 | 0.05 |
| Xenobiotics biodegradation and metabolism | Ethylbenzene degradation | 32 | ns | ns | 0.40 | ns | 0.05 | 0.003 | 0.05 | 0.06 | 0.04 | 0.08 |

^a^Statistical comparisons were made for those pathways that showed a relative abundance > 0.01% per intestinal site.

^b^Only significant (*P* ≤ 0.05) correlations are presented. * *P* ≤ 0.001.

^c^COG, Clusters of Orthologous Groups of proteins; KEGG, Kyoto Encyclopedia of Genes and Genomes; ns, not significant; RFI, residual feed intake; TFI, total feed intake; TBWG, total body weight gain; FCR, feed conversion ratio; SE, standard error; CI, confidence interval; Pctl, percentile.
