# Supplementary material for: Intestinal microbiota profiles associated with low and high residual feed intake in chickens across two geographical locations
Source: PLoS One. 2017 Nov 15;12(11):e0187766. doi: 10.1371/journal.pone.0187766 (PMC5687768; doi:10.1371/journal.pone.0187766)
Supplement: S6 Table — (DOCX) [file pone.0187766.s006.docx]

**S6 Table. Selected KEGG pathways correlating to feed efficiency and performance traits in male chickens across two geographical locations and by intestinal site.**

| COG Pathway^a-c^ | KEGG Pathway | n | RFI | TFI | TBWG | FCR | Mean | SE | Lower 95% CI | Upper 95% CI | 5th Pctl | 95th Pctl |
| --- | --- | --- | --- | --- | --- | --- | --- | --- | --- | --- | --- | --- |
| Ileum |  |  |  |  |  |  |  |  |  |  |  |  |
| Nucleotide metabolism | Nucleotide metabolism | 37 | ns | ns | ns | -0.34 | 0.12 | 0.01 | 0.11 | 0.14 | 0.05 | 0.18 |
| Membrane transport | Other ion-coupled transporters | 37 | ns | -0.33 | ns | ns | 1.69 | 0.03 | 1.63 | 1.76 | 1.37 | 2.00 |
|  |  |  |  |  |  |  |  |  |  |  |  |  |
| Ceca |  |  |  |  |  |  |  |  |  |  |  |  |
| Amino acid metabolism | Amino acid metabolism | 37 | ns | ns | ns | -0.44 | 0.20 | 0.01 | 0.19 | 0.22 | 0.15 | 0.27 |
| Amino acid metabolism | Amino acid related enzymes | 37 | ns | ns | ns | 0.33 | 1.66 | 0.01 | 1.64 | 1.69 | 1.51 | 1.77 |
| Amino acid metabolism | Tyrosine metabolism | 37 | -0.37 | ns | ns | -0.40 | 0.39 | 0.002 | 0.38 | 0.39 | 0.37 | 0.41 |
| Amino acid metabolism | Valine, leucine and isoleucine degradation | 37 | ns | ns | ns | -0.41 | 0.22 | 0.01 | 0.20 | 0.23 | 0.13 | 0.29 |
| Biosynthesis of other secondary metabolites | Novobiocin biosynthesis | 37 | ns | ns | -0.33 | ns | 0.14 | 0.002 | 0.13 | 0.14 | 0.11 | 0.16 |
| Biosynthesis of other secondary metabolites | Phenylpropanoid biosynthesis | 37 | ns | ns | ns | 0.34 | 0.12 | 0.005 | 0.11 | 0.13 | 0.06 | 0.17 |
| Carbohydrate metabolism | Ascorbate and aldarate metabolism | 37 | ns | ns | ns | -0.39 | 0.10 | 0.005 | 0.09 | 0.11 | 0.06 | 0.15 |
| Carbohydrate metabolism | Butanoate metabolism | 37 | ns | ns | ns | -0.43 | 0.82 | 0.01 | 0.80 | 0.85 | 0.72 | 0.96 |
| Carbohydrate metabolism | Citrate cycle (TCA cycle) | 37 | ns | ns | ns | -0.33 | 0.64 | 0.01 | 0.62 | 0.66 | 0.56 | 0.75 |
| Carbohydrate metabolism | Glyoxylate and dicarboxylate metabolism | 37 | ns | ns | ns | -0.40 | 0.56 | 0.01 | 0.54 | 0.58 | 0.44 | 0.67 |
| Carbohydrate metabolism | Inositol phosphate metabolism | 37 | ns | ns | 0.37 | -0.36 | 0.10 | 0.005 | 0.09 | 0.11 | 0.07 | 0.17 |
| Carbohydrate metabolism | Pentose and glucuronate interconversions | 37 | ns | ns | ns | -0.41 | 0.51 | 0.01 | 0.50 | 0.53 | 0.46 | 0.58 |
| Carbohydrate metabolism | Pyruvate metabolism | 37 | ns | ns | ns | -0.39 | 1.22 | 0.01 | 1.21 | 1.24 | 1.17 | 1.32 |
| Excretory system | Proximal tubule bicarbonate reclamation | 37 | ns | ns | ns | -0.36 | 0.02 | 0.001 | 0.02 | 0.02 | 0.01 | 0.03 |
| Folding, sorting and degradation | Chaperones and folding catalysts | 37 | ns | ns | ns | 0.35 | 1.02 | 0.01 | 1.00 | 1.03 | 0.93 | 1.08 |
| Folding, sorting and degradation | Protein export | 37 | ns | ns | ns | 0.39 | 0.68 | 0.005 | 0.67 | 0.69 | 0.63 | 0.73 |
| Folding, sorting and degradation | RNA degradation | 37 | ns | ns | ns | 0.40 | 0.50 | 0.003 | 0.50 | 0.51 | 0.47 | 0.54 |
| Folding, sorting and degradation | Restriction enzyme | 37 | ns | ns | ns | 0.34 | 0.22 | 0.005 | 0.21 | 0.23 | 0.18 | 0.29 |
| Lipid metabolism | Glycerophospholipid metabolism | 37 | ns | ns | -0.33 | ns | 0.59 | 0.01 | 0.58 | 0.61 | 0.51 | 0.65 |
| Lipid metabolism | Linoleic acid metabolism | 37 | ns | ns | 0.33 | ns | 0.08 | 0.005 | 0.07 | 0.09 | 0.06 | 0.15 |
| Lipid metabolism | Synthesis and degradation of ketone bodies | 37 | ns | ns | 0.34 | -0.40 | 0.05 | 0.002 | 0.05 | 0.06 | 0.02 | 0.07 |
| Membrane transport | ABC transporters | 37 | ns | ns | 0.35 | -0.33 | 4.40 | 0.06 | 4.27 | 4.52 | 4.01 | 5.26 |
| Membrane transport | Other ion-coupled transporters | 37 | ns | ns | ns | -0.34 | 1.29 | 0.02 | 1.26 | 1.32 | 1.09 | 1.45 |
| Metabolism of cofactors and vitamins | One carbon pool by folate | 37 | ns | ns | ns | 0.34 | 0.67 | 0.01 | 0.65 | 0.69 | 0.56 | 0.73 |
| Metabolism of cofactors and vitamins | Retinol metabolism | 37 | ns | ns | ns | -0.36 | 0.02 | 0.001 | 0.02 | 0.02 | 0.01 | 0.04 |
| Metabolism of other amino acids | beta-Alanine metabolism | 37 | -0.34 | ns | ns | ns | 0.13 | 0.004 | 0.12 | 0.14 | 0.09 | 0.18 |
| Metabolism of terpenoids and polyketides | Limonene and pinene degradation | 37 | ns | ns | ns | -0.33 | 0.10 | 0.004 | 0.09 | 0.11 | 0.07 | 0.15 |
| Signal transduction | Signal transduction mechanisms | 37 | ns | ns | ns | -0.34 | 0.61 | 0.005 | 0.60 | 0.62 | 0.57 | 0.66 |
| Signaling molecules and interaction | Cellular antigens | 37 | -0.34 | -0.37 | ns | ns | 0.01 | 0.001 | 0.01 | 0.01 | 0.003 | 0.03 |
| Translation | Aminoacyl-tRNA biosynthesis | 37 | ns | ns | ns | 0.34 | 1.45 | 0.02 | 1.41 | 1.48 | 1.27 | 1.61 |
| Translation | RNA transport | 37 | ns | ns | -0.37 | ns | 0.16 | 0.003 | 0.15 | 0.16 | 0.12 | 0.19 |
| Xenobiotics biodegradation and metabolism | Aminobenzoate degradation | 37 | ns | ns | 0.35 | -0.39 | 0.11 | 0.004 | 0.10 | 0.12 | 0.08 | 0.16 |
| Xenobiotics biodegradation and metabolism | Benzoate degradation | 37 | ns | ns | ns | -0.41 | 0.31 | 0.004 | 0.30 | 0.32 | 0.25 | 0.35 |
| Xenobiotics biodegradation and metabolism | Bisphenol degradation | 37 | ns | ns | 0.35 | -0.34 | 0.09 | 0.005 | 0.08 | 0.10 | 0.06 | 0.15 |
| Xenobiotics biodegradation and metabolism | Ethylbenzene degradation | 37 | ns | ns | 0.33 | -0.37 | 0.07 | 0.003 | 0.07 | 0.08 | 0.06 | 0.11 |
| Xenobiotics biodegradation and metabolism | Naphthalene degradation | 37 | ns | ns | ns | -0.44 | 0.17 | 0.003 | 0.17 | 0.18 | 0.15 | 0.21 |
| Xenobiotics biodegradation and metabolism | Xylene degradation | 37 | ns | ns | -0.34 | ns | 0.08 | 0.003 | 0.07 | 0.08 | 0.04 | 0.10 |
|  |  |  |  |  |  |  |  |  |  |  |  |  |
| Feces |  |  |  |  |  |  |  |  |  |  |  |  |
| Amino acid metabolism | Arginine and proline metabolism | 37 | ns | -0.40 | ns | ns | 1.12 | 0.03 | 1.05 | 1.19 | 0.64 | 1.43 |
| Amino acid metabolism | Histidine metabolism | 37 | -0.33 | -0.38 | ns | ns | 0.49 | 0.02 | 0.45 | 0.54 | 0.18 | 0.72 |
| Amino acid metabolism | Phenylalanine, tyrosine and tryptophan biosynthesis | 37 | ns | -0.39 | ns | ns | 0.66 | 0.03 | 0.60 | 0.73 | 0.08 | 0.97 |
| Amino acid metabolism | Valine, leucine and isoleucine biosynthesis | 37 | -0.40 | -0.44 | ns | ns | 0.67 | 0.03 | 0.61 | 0.72 | 0.26 | 0.89 |
| Carbohydrate metabolism | C5-Branched dibasic acid metabolism | 37 | ns | -0.39 | ns | ns | 0.29 | 0.01 | 0.26 | 0.32 | 0.03 | 0.38 |
| Carbohydrate metabolism | Pyruvate metabolism | 37 | ns | -0.39 | ns | ns | 1.22 | 0.02 | 1.18 | 1.26 | 1.07 | 1.35 |
| Drug resistance: Antimicrobial | beta-Lactam resistance | 37 | ns | 0.37 | ns | ns | 0.03 | 0.003 | 0.03 | 0.04 | 0.001 | 0.09 |
| Energy metabolism | Carbon fixation pathways in prokaryotes | 37 | -0.36 | -0.36 | ns | ns | 1.07 | 0.01 | 1.04 | 1.10 | 0.89 | 1.21 |
| Excretory system | Proximal tubule bicarbonate reclamation | 37 | 0.47 | 0.50 | 0.33 | ns | 0.04 | 0.003 | 0.03 | 0.04 | 0.01 | 0.06 |
| Folding, sorting and degradation | Chaperones and folding catalysts | 37 | ns | 0.36 | 0.40 | ns | 1.10 | 0.01 | 1.08 | 1.13 | 0.97 | 1.22 |
| Glycan biosynthesis and metabolism | Glycosyltransferases | 37 | ns | 0.37 | ns | ns | 0.48 | 0.02 | 0.45 | 0.51 | 0.28 | 0.63 |
| Lipid metabolism | Fatty acid biosynthesis | 37 | -0.34 | -0.46 | ns | ns | 0.51 | 0.01 | 0.48 | 0.54 | 0.34 | 0.66 |
| Membrane transport | Cell motility and secretion | 37 | ns | 0.48 | 0.33 | ns | 0.24 | 0.01 | 0.22 | 0.27 | 0.12 | 0.32 |
| Membrane transport | Phosphotransferase system (PTS) | 37 | ns | 0.35 | ns | ns | 1.09 | 0.07 | 0.95 | 1.24 | 0.27 | 2.06 |
| Metabolism of cofactors and vitamins | One carbon pool by folate | 37 | -0.33 | ns | ns | ns | 0.56 | 0.02 | 0.52 | 0.59 | 0.36 | 0.72 |
| Metabolism of cofactors and vitamins | Pantothenate and CoA biosynthesis | 37 | -0.35 | -0.44 | ns | ns | 0.60 | 0.01 | 0.57 | 0.63 | 0.45 | 0.79 |
| Nucleotide metabolism | Nucleotide metabolism | 37 | 0.35 | ns | ns | ns | 0.12 | 0.01 | 0.10 | 0.13 | 0.05 | 0.20 |
| Signaling molecules and interaction | Cellular antigens | 37 | 0.35 | ns | ns | ns | 0.04 | 0.004 | 0.03 | 0.04 | 0.01 | 0.08 |
| Transcription | Transcription related proteins | 37 | 0.41 | 0.37 | ns | ns | 0.03 | 0.003 | 0.02 | 0.03 | 0.001 | 0.06 |
| Translation | Translation proteins | 37 | -0.38 | ns | ns | ns | 1.04 | 0.02 | 1.00 | 1.08 | 0.80 | 1.20 |
| Transport and catabolism | Lysosome | 37 | ns | ns | -0.39 | ns | 0.01 | 0.002 | 0.01 | 0.01 | 0.001 | 0.03 |
| Xenobiotics biodegradation and metabolism | Drug metabolism - cytochrome P450 | 37 | ns | ns | ns | 0.03 | 0.08 | 0.01 | 0.07 | 0.10 | 0.01 | 0.17 |
| Xenobiotics biodegradation and metabolism | Fluorobenzoate degradation | 37 | 0.35 | ns | ns | ns | 0.02 | 0.004 | 0.01 | 0.03 | 0.0002 | 0.08 |

^a^Statistical comparisons were made for those pathways that showed a relative abundance > 0.01% per intestinal site.

^b^Only significant (*P* ≤ 0.05) correlations are presented.

^c^COG, Clusters of Orthologous Groups of proteins; KEGG, Kyoto Encyclopedia of Genes and Genomes; ns, not significant; RFI, residual feed intake; TFI, total feed intake; TBWG, total body weight gain; FCR, feed conversion ratio; SE, standard error; CI, confidence interval; Pctl, percentile.
