# Supplementary material for: Intestinal microbiota profiles associated with low and high residual feed intake in chickens across two geographical locations
Source: PLoS One. 2017 Nov 15;12(11):e0187766. doi: 10.1371/journal.pone.0187766 (PMC5687768; doi:10.1371/journal.pone.0187766)
Supplement: S7 Table — (DOCX) [file pone.0187766.s007.docx]

S7 Table. BLAST search results for most abundant operational taxonomic units (OTU) correlated to feed efficiency and performance traits in intestinal digesta and feces of broiler chickens raised at two geographical locations.

| OTU | Best BLAST hit^a^ | GenBank accession No. | Similarity [%] |
| --- | --- | --- | --- |
| OTU1 | *Escherichia coli* *Shigella flexneri* str. ATCC 29903 | GU968184.1 NR_026331.1 | 99.30 99.30 |
| OTU2 | *Turicibacter sanguinis* | AF349724.1 | 99.29 |
| OTU4 | *Lactobacillus crispatus* str. 214-1 | NZ_ADGR01000076.1 | 99.12 |
| OTU5 | *Clostridium* sp. str. FCB90-3 | AJ229251.1 | 86.45 |
| OTU6 | *Acetanaerobacterium elongatum* str. Z7 | AY487928.1 | 92.82 |
| OTU7 | *Anaerotruncus colihominis* str. 14565 | AJ315980.1 | 99.26 |
| OTU8 | *Lactobacillus crispatus* str. 214-1 | NZ_ADGR01000076.1 | 98.95 |
| OTU11 | *Streptococcus* subsp. *gallolyticus* str. UCN34 | FN597254.1 | 99.12 |
| OTU12 | *Clostridium* sp. str. WXC-8 | EU057612.1 | 87.41 |
| OTU14 | *Clostridium* sp. strain str. P6 | AY949857.1 | 86.68 |
| OTU15 | *Escherichia coli* *Shigella flexneri* str. ATCC 29903 | GU968184.1 NR_026331.1 | 99.30 99.30 |
| OTU17 | *Clostridium colicanis* str. DSM 13634 | AJ420008.1 | 86.31 |
| OTU18 | *Blautia* sp. str. GAM6-1 | HM626177.1 | 86.95 |
| OTU19 | *Clostridium* sp. str. FCB45 | AJ229248.1 | 87.57 |
| OTU22 | *Clostridium* sp. str. FCB90-3 | AJ229251.1 | 87.00 |
| OTU23 | *Clostridium cellulolyticum* H10 str. H10; ATCC 35319 | NC_011898.1 | 87.23 |
| OTU25 | *Clostridium* sp. str. FCB45 | AJ229248.1 | 87.20 |
| OTU29 | *Clostridium* sp. strain str. P6 | AY949857.1 | 86.86 |
| OTU31 | *Spiroplasma lampyridicola* str. ATCC 43206; PUP-1 | AY189134.1 | 82.56 |
| OTU32 | *Clostridium* sp. str. FCB45 | AJ229248.1 | 88.30 |
| OTU33 | *Ethanologenbacterium harbin* str. MD-12 | AY434718.1 | 92.31 |
| OTU35 | *Turicibacter sanguinis* | AF349724.1 | 99.12 |
| OTU38 | *Clostridium* sp. str. FCB90-3 | AJ229251.1 | 87.36 |
| OTU40 | *Turicibacter sanguinis* | AF349724.1 | 99.12 |
| OTU43 | *Eubacterium desmolans* | L34618.1 | 97.80 |
| OTU46 | *Faecalibacterium prausnitzii* str. M21/2 | NZ_ABED02000013.1 | 96.51 |
| OTU49 | *Clostridium* sp. str. P4-6 | FJ848364.1 | 88.12 |
| OTU52 | *Clostridium* sp. str. P4-6 | FJ848364.1 | 87.75 |
| OTU54 | *Enterobacter cloacae* subsp. *dissolvens* str. M425 | GU979185.1 | 99.12 |
| OTU55 | *Clostridium* sp. str. FCB90-3 | AJ229251.1 | 87.18 |
| OTU61 | *Clostridiaceae* str. 80Wd | AB078861.1 | 87.23 |
| OTU62 | *Streptococcus* subsp. *gallolyticus* str. UCN34 | FN597254.1 | 99.47 |
| OTU69 | *Clostridium cellulolyticum* H10 str. H10; ATCC 35319 | NC_011898.1 | 87.23 |
| OTU73 | *Acetanaerobacterium elongatum* str. Z7 | AY487928.1 | 93.39 |
| OTU75 | *Clostridium leptum* str. DSM 753T | AJ305238.1 | 94.30 |
| OTU77 | *Clostridium* sp. str. M62/1 | ACFX02000046.1 | 96.88 |
| OTU81 | *Enterobacter* sp. str. Nj-68 | AM491469.1 | 98.24 |
| OTU84 | *Eubacterium desmolans* | L34618.1 | 97.44 |
| OTU87 | *Clostridium* sp. strain str. P6 | AY949857.1 | 86.13 |
| OTU91 | *Heliorestis* sp. str. HR10B | EU908049.1 | 87.43 |
| OTU94 | *Clostridium* sp. str. FCB90-3 | AJ229251.1 | 97.00 |
| OTU99 | *Clostridium cellulolyticum* H10 str. H10; ATCC 35319 | NC_011898.1 | 87.04 |
| OTU103 | *Clostridium leptum* str. DSM 753T | AJ305238.1 | 95.22 |
| OTU104 | *Clostridium* sp. str. P4-6 | FJ848364.1 | 87.75 |
| OTU108 | *Clostridium cellulolyticum* H10 str. H10; ATCC 35319 | NC_011898.1 | 88.01 |
| OTU117 | *Clostridium* sp. str. M62/1 | ACFX02000046.1 | 97.79 |
| OTU125 | *Enterococcus faecalis* str. KLDS6.0609 | FJ607291.1 | 99.12 |
| OTU126 | *Eubacterium desmolans* | L34618.1 | 98.17 |
| OTU128 | *Clostridium* sp. str. FCB90-3 | AJ229251.1 | 85.71 |
| OTU131 | *Oscillibacter valericigenes* str. Sjm18-20 (= NBRC 101213) | AB238598.1 | 94.32 |
| OTU138 | *Escherichia coli* *Shigella flexneri* str. ATCC 29903 | GU968184.1 NR_026331.1 | 98.59 98.59 |
| OTU141 | *Clostridium* sp. str. YIT 12070 | AB491208.1 | 95.03 |
| OTU142 | *Clostridium* sp. str. FCB90-3 | AJ229251.1 | 87.00 |
| OTU143 | *Clostridium aldenense* str. RMA 9741 | DQ279736.1 | 94.14 |
| OTU145 | *Clostridium orbiscindens* str. AIP028.07 | EU541437.1 | 95.25 |
| OTU148 | *Escherichia coli* *Shigella flexneri* str. ATCC 29903 | GU968184.1 NR_026331.1 | 99.12 99.12 |
| OTU150 | *Clostridium* sp. str. M62/1 | ACFX02000046.1 | 98.35 |
| OTU151 | *Clostridium leptum* str. DSM 753T | AJ305238.1 | 94.12 |
| OTU152 | *Clostridium* sp. str. FCB90-3 | AJ229251.1 | 87.04 |
| OTU157 | *Oscillibacter valericigenes* str. Sjm18-20 (= NBRC 101213) | AB238598.1 | 94.14 |
| OTU161 | *Clostridiaceae* str. 80Wd | AB078861.1 | 86.50 |
| OTU164 | *Clostridium asparagiforme* str. DSM 15981 | ACCJ01000522.1 | 95.59 |
| OTU165 | *Spiroplasma lampyridicola* str. ATCC 43206; PUP-1 | AY189134.1 | 81.32 |
| OTU166 | *Turicibacter sanguinis* | AF349724.1 | 97.15 |
| OTU167 | *Dehalobacterium formicoaceticum* str. DMC | X86690.1 | 91.07 |
| OTU173 | *Clostridium leptum* str. DSM 753T | AJ305238.1 | 92.65 |
| OTU174 | *Turicibacter sanguinis* | AF349724.1 | 99.29 |
| OTU179 | *Clostridium* sp. str. FCB45 | AJ229248.1 | 87.20 |
| OTU182 | *Clostridium spiroforme* str. DSM 1552 | NZ_ABIK02000013.1 | 99.47 |
| OTU188 | *Clostridium* sp. str. FCB45 | AJ229248.1 | 88.28 |
| OTU190 | *Eubacterium desmolans* | L34618.1 | 96.17 |
| OTU192 | *Clostridium* sp. str. FCB90-3 | AJ229251.1 | 87.36 |
| OTU203 | *Anaerostipes* sp. str. 35-7 | FJ947528.1 | 97.61 |
| OTU205 | *Hespellia porcina* str. PPC80 | AF445239.2 | 95.96 |
| OTU207 | *Hespellia porcina* str. PPC80 | AF445239.2 | 95.59 |
| OTU209 | *Turicibacter sanguinis* | AF349724.1 | 97.53 |
| OTU210 | *Acetanaerobacterium elongatum* str. Z7 | AY487928.1 | 89.28 |
| OTU212 | *Enterobacter cloacae* Nr. 3 | Y17665.1 | 99.12 |
| OTU215 | *Clostridium hylemonae* str. CT-35 | AB117570.1 | 95.78 |
| OTU217 | *Clostridium* sp. str. FCB90-3 | AJ229251.1 | 86.63 |
| OTU220 | *Escherichia coli* *Shigella flexneri* str. ATCC 29903 | GU968184.1 NR_026331.1 | 96.66 96.66 |
| OTU224 | *Clostridium* sp. str. P4-6 | FJ848364.1 | 87.02 |
| OTU228 | *Spiroplasma lampyridicola* str. ATCC 43206; PUP-1 | AY189134.1 | 85.05 |
| OTU233 | *Clostridium* sp. str. P4-6 | FJ848364.1 | 87.02 |
| OTU237 | *Anaerotruncus colihominis* str. WAL 14565; DSM 17241 | NR_027558.1 | 95.21 |
| OTU238 | *Eubacterium desmolans* | L34618.1 | 97.99 |
| OTU252 | *Eubacterium coprostanoligenes* str. HL | HM037995.1 | 93.21 |
| OTU253 | *Turicibacter sanguinis* | AF349724.1 | 97.33 |
| OTU259 | *Shigella sonnei* str. 136 | GQ259886.1 | 94.38 |
| OTU274 | *Ruminococcus flavefaciens* str. JF1 | AY445601.1 | 88.44 |
| OTU276 | *Turicibacter sanguinis* | AF349724.1 | 96.64 |
| OTU281 | *Clostridium leptum* str. DSM 753T | AJ305238.1 | 95.4 |
| OTU285 | *Clostridium* sp. str. EBR-02E-0045 | AB186359.1 | 87.18 |
| OTU288 | *Turicibacter sanguinis* | AF349724.1 | 95.08 |
| OTU289 | *Clostridium* sp. FG4 | AB207248.1 | 86.5 |
| OTU292 | *Clostridiaceae* str. 80Wd | AB078861.1 | 87.04 |
| OTU294 | *Shigella* sp. str. 4104 | FJ405328.1 | 94.20 |
| OTU295 | *Clostridium propionicum* str. DSM 1682 | X77841.1 | 94.85 |
| OTU298 | *Escherichia coli* *Shigella flexneri* str. ATCC 29903 | GU968184.1 NR_026331.1 | 99.12 99.12 |
| OTU308 | *Clostridium* sp. str. 6-29 | FJ808604.1 | 84.49 |
| OTU323 | *Turicibacter sanguinis* | AF349724.1 | 96.47 |
| OTU325 | *Clostridium* sp. str. 6-29 | FJ808604.1 | 85.77 |
| OTU329 | *Clostridium* sp. str. M62/1 | ACFX02000046.1 | 99.08 |
| OTU341 | *Clostridium* sp. str. M62/1 | ACFX02000046.1 | 96.69 |
| OTU343 | *Anaerotruncus colihominis* str. WAL 14565; DSM 17241 | NR_027558.1 | 98.53 |
| OTU346 | *Anaerotruncus colihominis* str. WAL 14565; DSM 17241 | NR_027558.1 | 94.11 |
| OTU348 | *Ruminococcus* sp. str. 14531 | AJ315979.1 | 98.35 |
| OTU349 | *Sporobacter termitidis* str. SYR | Z49863.1 | 93.60 |
| OTU352 | *Turicibacter sanguinis* | AF349724.1 | 96.47 |
| OTU362 | *Clostridium* sp. FG4 | AB207248.1 | 85.40 |
| OTU368 | *Ruminococcus* sp. str. MLG080-3 | AY653234.1 | 95.23 |
| OTU388 | *Clostridium cellulolyticum* H10 str. H10; ATCC 35319 | NC_011898.1 | 87.41 |
| OTU392 | *Clostridium cellulolyticum* H10 str. H10; ATCC 35319 | NC_011898.1 | 87.41 |
| OTU415 | *Clostridium* sp. str. YIT 12070 | AB491208.1 | 93.92 |
| OTU437 | *Clostridium* sp. str. 6-29 | FJ808604.1 | 86.50 |
| OTU456 | *Photorhabdus luminescens* PDBC EPN6.71 | AY597525.2 | 90.86 |
| OTU459 | *Clostridium cellulolyticum* H10 str. H10; ATCC 35319 | NC_011898.1 | 87.41 |
| OTU466 | *Clostridium* sp. str. SDG-Mt85-3Db | DQ100445.1 | 89.77 |
| OTU469 | *Clostridium* sp. str. FCB90-3 | AJ229251.1 | 86.24 |
| OTU470 | *Clostridium* sp. str. P4-6 | FJ848364.1 | 87.75 |
| OTU482 | *Clostridium* sp. str. P4-6 | FJ848364.1 | 87.00 |
| OTU500 | *Clostridium cellulolyticum* H10 str. H10; ATCC 35319 | NC_011898.1 | 88.32 |
| OTU504 | *Clostridium hylemonae* str. CT-35 | AB117570.1 | 95.59 |
| OTU507 | *Clostridium cellulolyticum* H10 str. H10; ATCC 35319 | NC_011898.1 | 86.86 |
| OTU515 | *Clostridium* sp. FG4 | AB207248.1 | 86.68 |
| OTU519 | *Ruminococcus lactaris* str. ATCC 29176 | ABOU02000049.1 | 95.59 |
| OTU520 | *Clostridium* sp. str. FCB45 | AJ229248.1 | 87.75 |
| OTU553 | *Clostridium* sp. strain str. P6 | AY949857.1 | 85.04 |
| OTU574 | *Eubacterium* sp. str. WAL 17363 | GQ461729.1 | 97.32 |
| OTU610 | *Clostridium leptum* str. DSM 753T | AJ305238.1 | 93.57 |
| OTU619 | *Pseudidiomarina* sp. str. 908087 | EU600203.2 | 99.12 |
| OTU634 | *Clostridium* sp. str. 6-29 | FJ808604.1 | 86.50 |
| OTU654 | *Ruminococcus* sp. str. CCUG 37327 | AJ318864.1 | 86.11 |
| OTU725 | *Clostridium hylemonae* str. CT-35 | AB117570.1 | 95.78 |

^a^Greengenes database (greengenes.lbl.gov/cgi-bin/nph-index.cgi); last accessed 4/21/2017.
