# Supplementary material for: Intestinal microbiota profiles associated with low and high residual feed intake in chickens across two geographical locations
Source: PLoS One. 2017 Nov 15;12(11):e0187766. doi: 10.1371/journal.pone.0187766 (PMC5687768; doi:10.1371/journal.pone.0187766)
Supplement: S8 Table — (DOCX) [file pone.0187766.s008.docx]

**S8 Table. Pearson’s correlations between selected bacterial genera and KEGG pathways in cecal digesta associated with the residual feed intake in male and female chickens across two geographical locations.**

|  | KEGG pathways |  |  |  |  | KEGG pathways |  |
| --- | --- | --- | --- | --- | --- | --- | --- |
| Genus^a,b^ | Cellular antigens | Tyrosine metabolism | beta-Alanine metabolism |  | Genus | Lysine biosynthesis | Phenylalanine, tyrosine and tryptophan biosynthesis |
| Males |  |  |  |  | Females |  |  |
| Unclassified *Clostridiales* 1 | ns | ns | ns |  | Unclassified *RF39* | ns | ns |
| *Lactobacillus* | ns | ns | ns |  |  |  |  |
| Unclassified *Ruminococcaceae* | ns | ns | ns |  |  |  |  |
| *Ruminococcus* | ns | ns | 0.35 |  |  |  |  |
| *Coprococcus* | ns | ns | ns |  |  |  |  |

^a^ Statistical comparisons were made for bacterial genera and KEGG pathways that were associated with chicken’s residual feed intake.

^b^ Only significant (*P* ≤ 0.05) correlations are presented.

^c^ KEGG, Kyoto Encyclopedia of Genes and Genomes; ns, not significant.
