# Supplementary material for: Intestinal microbiota profiles associated with low and high residual feed intake in chickens across two geographical locations
Source: PLoS One. 2017 Nov 15;12(11):e0187766. doi: 10.1371/journal.pone.0187766 (PMC5687768; doi:10.1371/journal.pone.0187766)
Supplement: S9 Table — (DOCX) [file pone.0187766.s009.docx]

**S9 Table. Pearson’s correlations between selected bacterial genera and KEGG pathways in feces associated with the residual feed intake in male chickens across two geographical locations.**

| Genus | Carbon fixation pathways in prokaryotes | Cellular antigens | Fatty acid biosynthesis | Fluoro-benzoate degradation | Histidine metabolism | Nucleotide metabolism | One carbon pool by folate | Pantothenate and CoA biosynthesis | Proximal tubule bicarbonate reclamation | Transcription related proteins | Translation proteins | Valine, leucine and isoleucine biosynthesis |
| --- | --- | --- | --- | --- | --- | --- | --- | --- | --- | --- | --- | --- |
| *Lactobacillus* | -0.53 | 0.64 | -0.54 | ns | -0.64 | ns | ns | -0.58 | 0.58 | ns | ns | -0.76 |
| Unclassified *RF39* | ns | -0.34 | ns | ns | 0.44 | -0.42 | 0.42 | ns | -0.40 | -0.44 | 0.42 | 0.39 |
| Unclassified *Clostridiales 2* | ns | ns | ns | ns | ns | ns | ns | ns | ns | ns | ns | ns |
| Unclassified *Lachnospiraceae 1* | 0.44 | -0.52 | 0.48 | -0.37 | 0.63 | -0.57 | 0.54 | 0.52 | -0.52 | -0.61 | 0.53 | 0.57 |
| *Acinetobacter* | ns | 0.42 | ns | 0.68 | ns | ns | ns | -0.35 | 0.45 | ns | -0.43 | ns |
| *Pseudomonas* | ns | ns | ns | 0.55 | ns | ns | ns | ns | ns | ns | ns | ns |
| *Dorea* | 0.36 | -0.37 | 0.40 | -0.34 | 0.54 | -0.54 | 0.51 | 0.44 | -0.45 | -0.55 | 0.46 | 0.49 |

^a^ Statistical comparisons were made for bacterial genera and KEGG pathways that were associated with chicken’s residual feed intake.

^b^ Only significant (*P* ≤ 0.05) correlations are presented.

^c^ KEGG, Kyoto Encyclopedia of Genes and Genomes; ns, not significant.
