# Supplementary material for: Intestinal microbiota profiles associated with low and high residual feed intake in chickens across two geographical locations
Source: PLoS One. 2017 Nov 15;12(11):e0187766. doi: 10.1371/journal.pone.0187766 (PMC5687768; doi:10.1371/journal.pone.0187766)
Supplement: S10 Table — (DOCX) [file pone.0187766.s010.docx]

**S10 Table. Pearson’s correlations between selected bacterial genera and KEGG pathways in feces associated with the residual feed intake in female chickens across two geographical locations.**

| Females | C5-Branched dibasic acid metabolism | Ion channels | Lipid biosynthesis proteins | Lipoic acid metabolism | Lysine biosynthesis | Novobiocin biosynthesis | Phenylalanine, tyrosine and tryptophan biosynthesis | Tropane, piperidine and pyridine alkaloid biosynthesis |
| --- | --- | --- | --- | --- | --- | --- | --- | --- |
| *Lactobacillus* | -0.67 | ns | ns | ns | ns | -0.45 | -0.52 | -0.45 |
| Unclassified *Clostridiales 2* | ns | ns | ns | ns | ns | ns | ns | ns |
| *Acinetobacter* | ns | ns | ns | ns | ns | ns | ns | ns |
| Unclassified *Lachnospiraceae 2* | ns | -0.53 | 0.49 | -0.40 | 0.50 | ns | 0.46 | ns |
| *Pseudomonas* | ns | ns | ns | ns | ns | ns | ns | ns |

^a^ Statistical comparisons were made for bacterial genera and KEGG pathways that were associated with chicken’s residual feed intake.

^b^ Only significant (*P* ≤ 0.05) correlations are presented.

^c^ KEGG, Kyoto Encyclopedia of Genes and Genomes; ns, not significant.
